# A Scoping Review of Behavioural Studies on Heated Tobacco Products

**DOI:** 10.7759/cureus.65773

**Published:** 2024-07-30

**Authors:** Ian M Fearon, Sarah F Cordery, Martin Fitzpatrick, Sarah Weaver, Matthew Stevenson, Erika Grandolfo, Layla Malt, Keith Thompson, Thomas Nahde

**Affiliations:** 1 Scientific Research, whatIF? Consulting Ltd., Harwell, GBR; 2 Group Science and Regulatory Affairs, Imperial Brands PLC, Bristol, GBR; 3 Scientific Consultant, Elucid8 Holdings Ltd., Newtownabbey, GBR; 4 Group Science and Regulatory Affairs, Imperial Brands Reemtsma, Hamburg, DEU

**Keywords:** scoping review, population health, tobacco harm reduction, cigarette smoking, heat-not-burn, tobacco heating products, heated tobacco products

## Abstract

Heated tobacco products (HTPs) are electronic devices that heat tobacco sticks to temperatures much lower than those which cause pyrolysis and combustion in cigarettes. While this electrical heating causes the formation of an inhalable aerosol which contains nicotine, the aerosol from HTPs contains significantly fewer and lower levels of the harmful and potentially harmful chemicals found in cigarette smoke. As a result, HTP use potentially conveys reduced risks to health compared to cigarette smoking. While this relative reduction in individual health risk is becoming clearer, what is less certain is the impact of HTPs on overall population‑level health, taking into account both the potential positive impact on adult smokers who completely switch to using HTPs and any unintended impacts such as use by tobacco non‑users and particularly by youth. The aim of this scoping review was to collate and evaluate the published scientific evidence to date, with a cut‑off of 1 January 2024, investigating the impact of HTPs on population‑level health. This evaluation suggests that HTP use is almost exclusively observed among those with a history of cigarette smoking, and there is a growing body of evidence for the ability of HTPs to provide support for adult smokers to transition away from cigarette smoking, in the absence of any significant “gateway” into tobacco use initiation. Many studies have reported a significant degree of dual use of cigarettes and HTPs, and efforts to assess the reasons for such patterns of use, whether these provide overall exposure reductions, and whether dual use acts as a bridge towards a complete transition away from cigarette smoking, requires further investigation. In addition, correction of the widespread and increasing misperceptions of HTPs among adult smokers is recommended to promote HTP uptake as a potentially less harmful alternative to smoking in this population.

## Introduction and background

Smoking is a cause of serious diseases in smokers, including lung cancer, heart disease, and emphysema. It has been estimated that over one billion smokers consume over five trillion cigarettes each year, and smoking is estimated to be responsible for eight million deaths globally each year [1] and is predicted to potentially lead to a billion deaths in the 21st century [2]. While many complex mechanisms underpin the development of smoking‑related diseases, the overall initiating event is the inhalation of many thousands of chemicals in cigarette smoke, which are principally produced by the processes of pyrolysis and combustion of tobacco [3]. The greatest risk of smoking-related disease stems from burning tobacco and inhaling the smoke. Tobacco smoke contains around 7,000 chemicals, of which 100 are classified by public health experts as ‘harmful and potentially harmful constituents' (HPHCs), linking them to causing or potentially causing smoking-related disease [4]. Although quitting smoking remains the optimal method for smokers to prevent damage to their health [5], for those who are uninterested or unwilling to quit, a tobacco harm reduction (THR) strategy has been suggested as a potentially advantageous approach to mitigate the health risks associated with cigarette smoking, benefiting both individuals and public health [6,7]. Defined as minimizing harms and decreasing total mortality and morbidity, one of the main principles of THR is that the health burden of cigarette smoking can be reduced by allowing smokers access to potentially less harmful alternatives to combustible tobacco products without completely eliminating tobacco and/or nicotine use [8]. Such alternatives, whilst not entirely risk‑free, would either reduce or eliminate exposure to HPHCs and other toxicants and potentially therefore reduce the incidence of smoking-related disease [7-9].

Heated tobacco products (HTPs) heat a tobacco substrate to release an inhalable nicotine-containing aerosol. Commercially available products include electronically heated HTPs, which heat the tobacco using resistive or induction technologies, and, less commonly, carbon-heated HTPs, which heat the tobacco using a lit carbon heat source. While HTP aerosols contain nicotine and flavour aromas from tobacco, the levels of HPHCs and other toxicants in their aerosols are greatly reduced compared to cigarette smoke with some smoke toxicants completely absent, and a small number of additional toxicants found in HTP aerosol that are not present in cigarette smoke are at levels of no toxicological concern [10-14]. Given this differential aerosol toxicant load, toxicological assessments have shown that the aerosol from HTPs is less cytotoxic, genotoxic, mutagenic, and carcinogenic, than cigarette smoke and also possesses lower activity in other in vitro and in vivo assays [13-30]. Furthermore, clinical studies in which smokers have completely switched to using HTPs have demonstrated reduced exposure to tested panels of smoke toxicants, in some cases to levels approaching those seen with complete abstinence from tobacco/nicotine product use [31-37]. This can lead to favourable changes in biomarkers of potential harm, which are considered to be indicators of future disease risk [31,38-41]. These findings support that HTPs have harm reduction potential, at least at the individual level, for smokers who completely switch to using them, which is supported by reviews of the literature concerning the individual health impact of HTPs [19,42-46].

While HTPs appear to offer individual‑level THR potential for smokers who completely switch, the potential population‑level health impact of HTPs is somewhat ambiguous. At the population level, various factors are important in determining the potential contribution of HTPs to THR. These include the ability of HTPs to appeal to current smokers, who are otherwise uninterested or unwilling to quit smoking, and to facilitate their complete transitioning away from cigarette smoking, as well as whether HTPs are appealing to tobacco non‑users and their potential use as means of initiating tobacco product use [47,48]. To fulfil THR potential on a population level, next-generation products including HTPs must be acceptable for adult smokers but, importantly, not attract unintended populations such as never smokers, ex-smokers, or youth [49].

The aim of this review therefore is to collate and critically assess the scientific literature to date concerning the population‑level impact of electronically heated HTPs and to use this information to gain a fuller understanding of their THR potential.

## Review

Methods

Information Source and Search Terms

An interrogation of the PubMed database (www.pubmed.ncbi.nlm.nih.gov) was conducted in January 2024 to identify all potentially relevant peer‑reviewed journal articles relating to HTPs using the following search terms: "heated tobacco" OR "tobacco heating" OR “heat tobacco” OR "tobacco heating system" OR "heated tobacco product" OR “heat‑not‑burn” OR “heated cigarette” OR "iqos" OR "glo" OR "pulze" OR "ploom".

The search was restricted to a date range from the inception of the PubMed database to 1 January 2024. This resulted in a list of 3,056 articles, which were manually screened using the article title, abstract, and where necessary the article text. This resulted in the identification of 157 articles relevant to the topics of this scoping review.

Eligibility Criteria

Articles were selected if they were related to one or more of the following topics: awareness; risk perceptions and beliefs; intentions to use and susceptibility of use; user demographics and smoking status; drivers of use or reasons for use; impact on smoking reductions or cessation; and prevalence of use. Articles were excluded if they related to the following topics: study methodologies and protocols which did not present original data; impact on COVID‑19 infection or changes in product use due to COVID‑19; clinical studies (e.g., health effect/biomarker studies, abuse liability assessments, cue reactivity, topography); emissions chemistry; abuse liability and dependence; toxicology studies; animal studies; secondhand exposure (e.g., environmental tobacco smoke or indoor air quality); excise and taxation; impact of hypothetical messaging; health warnings; advertising/marketing strategies and exposure to marketing; indirect perceptions; exposure, articles not written in English; review articles including systematic reviews or meta‑analyses; opinion pieces and letters to Editors; errata; papers discussing the regulatory environment; assessment of social media interest or content and news/media coverage; products other than electronically HTPs (e.g., waterpipe/shisha, carbon HTPs, and electronically heated products which heat a herbal and not a tobacco substrate); and articles providing an analysis of internal industry documents or activities. No restrictions were applied for specific HTPs, but studies examining individual components of HTPs (e.g., additives, flavourings) were not considered relevant. 

Categorisation of Articles

All eligible articles were categorised according to their subject matter into one of the following: perceptions and beliefs including risk perceptions; youth, adolescent, and young adult awareness and use; adult awareness and use; drivers of use and reasons for use; impacts on cigarette smoking, including smoking reductions, cessation, and relapse; and population modelling. These categories are used in the results section of this review. For papers spanning more than one category (e.g., youth and adult use, or perceptions and prevalence of use), the most relevant single category was chosen. In the results section, each paper is described and discussed only once, and cross‑references to other categories are made where it was deemed appropriate.

Critical Appraisal

This scoping review was drafted in accordance with the guidelines proposed by the Preferred Reporting Items for Systematic Reviews and Meta‑Analyses Extension for Scoping Reviews (PRISMA‑ScR) checklist. The evidence‑based reporting system for systematic reviews set forth by the PRISMA group advises that conducting a formal assessment of individual study methodological quality is not a typical feature of a scoping review [50]. As such, no formal assessment of quality was included in this paper.

Results

**Figure 1:**
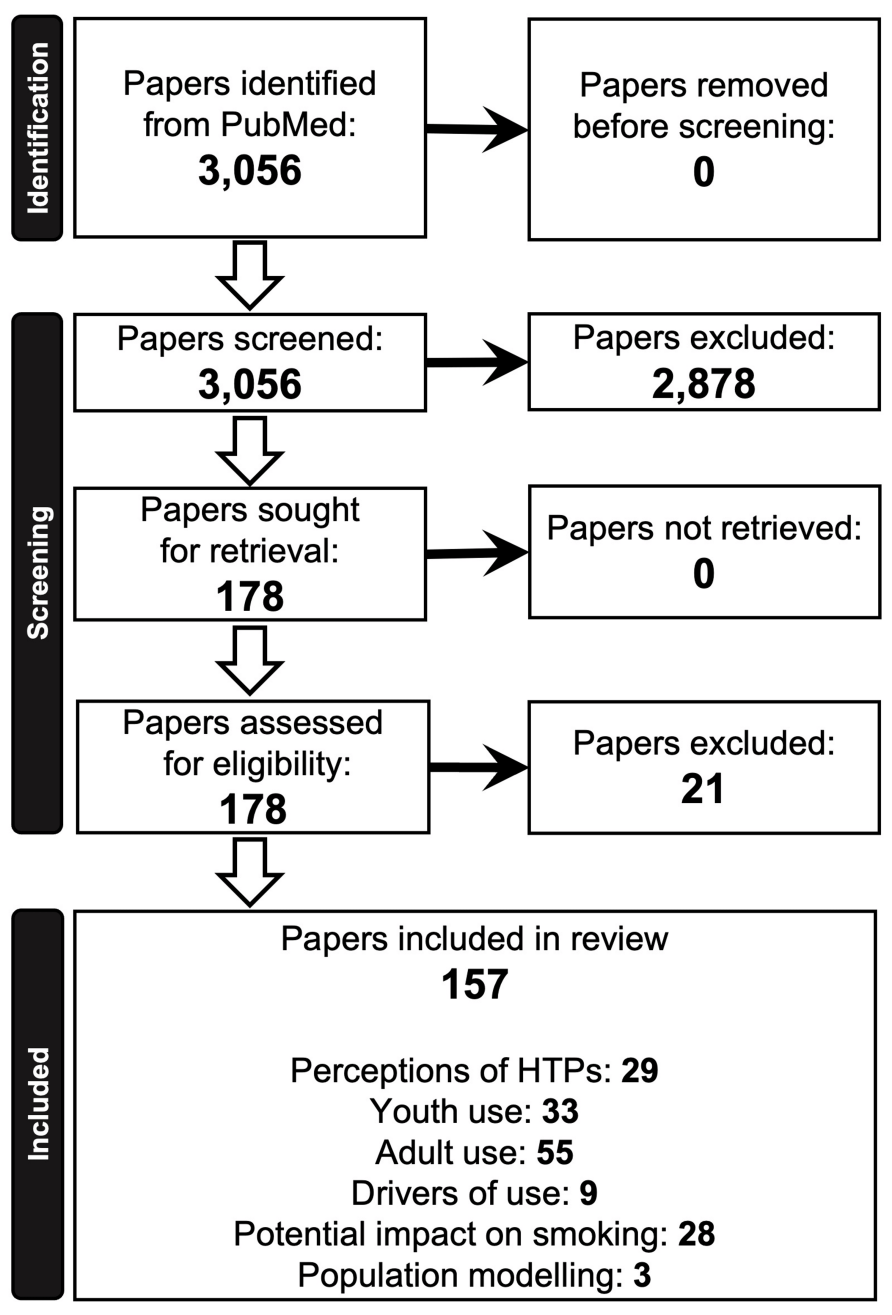
Flow diagram depicting the process of paper selection and topic areas of the identified papers.

Information detailing the number of articles identified, screened, and discussed in this review is presented in Figure 1. The PubMed database search identified 3,056 potentially eligible published articles. Following the title, abstract, and where necessary article text screening and assessment of eligibility, a total of 157 articles were deemed eligible for inclusion. Of these, 29 were primarily related to perceptions of HTPs, 33 were primarily related to youth use, 55 were primarily related to adult use, nine were primarily related to drivers of use, 28 were primarily related to the potential impacts of HTP use on cigarette smoking, and three were primarily related to modelling of the potential population‑level impact of HTPs.

Perceptions and Beliefs, Including Risk Perceptions

Perceptions of the harmfulness of nicotine‑containing products, both absolute and relative to cigarette smoking, are potential determinants of future use of that product [51]. For example, smokers who perceive that nicotine replacement therapy (NRT) products are just as harmful as cigarettes, or who are unsure about the relative risk, have been found to be less likely to have used NRT in the past or to consider using NRT in the future quit attempts [52]. In addition, those with NRT safety concerns use such products less frequently and for shorter periods of time [52]. Furthermore, beliefs surrounding the relative efficacy of NRT (i.e., its ability to help smokers stop smoking) are a potential determinant of both NRT uptake and compliant use [52,53]. The same principles apply to non‑pharmaceutical nicotine‑containing products, and many studies have reported that tobacco and nicotine product risk perceptions, in areas such as the potential to cause harm or addictiveness, are associated with and predict tobacco use behaviour [54,55].

Perceptions of the IQOS HTP

Given the link between perceptions and use, either actual or intended, many studies have specifically examined perceptions of HTPs in various countries in which they are marketed, and a number of those studies have assessed perceptions of IQOS, one of several commercially available HTPs. In the earliest assessment, Hair et al. conducted expert interviews with professionals working in youth culture and youth/young adult tobacco and e‑cigarette use to gain insight into how IQOS might fit into cultures and markets [56]. The findings from these interviews, conducted in Japan and Switzerland, were then used to guide focus group studies. Data from the expert interviews identified two main concepts of potential IQOS use: 'freedom”, in terms of a tension between using technology as freedom of expression, to pursue emotional desires, and to set yourself apart; and 'control”, in terms of controlling your body, organising your life and uncovering the processes behind the goods you consume. In the Japanese focus groups, participants found IQOS to be a clean, chic, and luxurious product, and using it when socialising with groups of non‑smokers where cigarette smoking was disallowed could impact smoke‑free social situations. Some negative perceptions were reported, such as the cumbersome, bulky size of the device and its charger, and the impact of these on portability. Price was also perceived as a negative attribute and a barrier to use among Japanese youth/young adults. Charging, as well as needing to clean the device after use, was identified as negative perceptions among the Swiss participants. IQOS generally did not appear to fit well into Swiss youth/young adult culture. Certainly, smoking was seen as rebellious, whereas the use of IQOS did not fit into this aspect of self‑expression. It is also noteworthy that, in Switzerland and Japan, lower levels of satisfaction were reported for IQOS compared to cigarettes.

Using data from the 2018 International Tobacco Control Japan-Canada Heated Tobacco Products (ITC‑JCH) internet survey study, focussing on Canadian adults aged 20 and older with different smoking statuses, Sutanto et al. [57] assessed perceptions of IQOS subsequent to its launch in Canada in 2017. Among the 268 participants, less than half (48%) of participants perceived IQOS use as being less harmful than cigarette smoking. Both exclusive e‑cigarette users and dual users of e‑cigarettes and cigarettes, but not exclusive smokers, had higher odds of perceiving IQOS as less harmful than cigarettes compared to non‑users. The reasons for the general misperceptions were not reported or discussed, but these could act as a barrier to transitioning away from cigarette smoking among current smokers as has been reported for other reduced-risk smoking alternatives [52,58-61]. Relative risk perceptions of IQOS and cigarettes have been examined further, using cross‑sectional data from 10,037 adult IQOS users in Germany, Italy, and Japan, in different survey study waves between 2016 and 2020 [62]. Overall, and in contrast to Sutanto et al. [57], the perception of the risk of cigarette smoking was higher than that of IQOS use. While absolute risk perceptions of cigarettes remained stable over time across all countries and were similar between countries, absolute risk perceptions of IQOS increased over time in Japan, but not in Germany and Italy, and this perhaps highlights between-country and cultural differences in IQOS risk perceptions. When assessed as a relative risk of smoking compared with IQOS use, risk perceptions were stable over time in Germany and Italy but increased over time in Japan, which appears to be due to the increase in perceived risk of IQOS use over time in Japan, while perceived risks of smoking remained stable. Gender was reported to play a role in risk perceptions. For example, relative risk perception was slightly lower among German males than females, while the opposite was observed in Japan. IQOS use behaviour also played a role, with predominant and more frequent IQOS users perceiving a lower relative risk of IQOS use compared with cigarette smoking.

In a further, qualitative, assessment of perceived risks among adult current and former IQOS users in the United Kingdom (UK) [63], IQOS was generally perceived as less harmful than smoking but not risk‑free, although there was great uncertainty in terms of large proportions of respondents reporting ‘don’t know’ regarding the harms associated with IQOS use. In this study, factors which were reported to influence risk perceptions were consolidated into six themes: dominance of manufacturer claims, although there was skepticism about claims coming from the industry; limited independent and long‑term research leading to uncertainty about risk; appearance of HTP tobacco stick packaging conveyed reduced harm because packets were ‘pretty’ and had no graphic/specific health warnings, although written warnings conveyed some harm; the process of heating, compared with burning, tobacco was perceived to produce fewer harmful chemicals; improvements in participants’ own physical health and personal appearance reduced perceptions of harm; and differences in sensory experiences (taste, sight, smell) when using IQOS compared with smoking reduced perceptions of harm, while ‘black’ deposits inside IQOS led to perceptions of some harm. Additionally, the reduced volume and smell of IQOS emissions compared to cigarette smoke also reduced perceptions of harm to non‑users exposed to the emissions. In another qualitative interview study conducted among 30 adult current or former users of IQOS in the UK [64], participants expressed confusion on how to describe IQOS use, which appeared to be amplified by similarities with the physicality and rituals of cigarette smoking, as well as both similarities and differences compared to e‑cigarette use. Similar to studies by Duan et al. [65] and Kim et al. [66] described later in this section, these similarities led to participants’ positioning of IQOS use as a hybrid of both cigarette smoking and e‑cigarette use [64]. 

Mays et al. [67] conducted an online survey study in which 1,328 young adult (aged 18-30) cigarette smokers and non‑smokers viewed an IQOS advertisement with claim variations which were either developed by Phillip Morris International (PMI) and authorised by the US Food and Drug Administration (FDA) as part of a modified risk tobacco product (MRTP) determination or were hypothetical claims derived by the study authors. The study assessed the perceived credibility and effectiveness of the health or risk message for discouraging IQOS use, perceived harms, efficacy beliefs, and IQOS use intentions. Smokers reported significantly higher perceived credibility, lower perceived effectiveness, higher efficacy beliefs about transitioning to IQOS use, and higher intentions to use IQOS, than non‑smokers. Among smokers, health warnings significantly increased perceived credibility and effectiveness, but claims did not affect intention outcomes. Among non‑smokers, warnings and claims increased perceived credibility, and warnings increased perceived effectiveness. When assessing the FDA‑authorised exposure claims, these increased non‑smokers’ intentions to use IQOS. Intriguingly, among non‑smokers, advertisements with a reduced harm claim produced greater perceived harms of IQOS relative to cigarettes compared to advertisements with no claims, highlighting the complexity of consumer‑facing claims development.

An interesting qualitative approach to assessing IQOS perceptions was described in a study by Kim et al. [66] in which 33 young adult (aged 18-29) Californian tobacco and nicotine (mainly cigarette and e‑cigarette) users were asked to ‘unbox’ a new IQOS device and tobacco sticks. Participants were also presented with IQOS marketing materials and were asked to narrate their impressions and opinions [66]. Similar to the findings of Hair et al. [56] described previously, this study identified multiple attributes which influenced IQOS appeal, including its sleek electronic design, use of novel technology incorporating a new device with traditional tobacco, perceived harmfulness, complexity, and high cost [66]. During unboxing, more than half of the participants discussed their perceptions of the potential harmfulness of IQOS, which many believed could be less harmful than cigarettes, and one participant alluded to the lack of burning as presenting less harm. In contrast, however, the similarity of the HTP tobacco sticks to cigarettes increased participants’ perceptions of potential harm. Regarding interest in using IQOS, 24 participants showed at least some interest, including seven who expressed low or moderate interest in trying it, 13 who explicitly expressed interest in trying it, three who were interested in buying it, and one who had already bought it. Of the 16 participants who expressed interest in either buying or trying IQOS, all but two were current smokers, mostly dual users. By contrast, among the low/no interest group, only half were still smoking cigarettes. Some participants also commented on the fact that IQOS resembles a hybrid of an e‑cigarette and a cigarette, and potentially this portrays a new method of using an old, harmful product, which could increase perceptions of risk and decrease the likelihood of use. This theme was also identified by Duan et al. [65], who analysed data from a mixed qualitative/quantitative survey study conducted among 2,470 young adults aged between 18 and 34 in the US in 2020 and 2021. Analysis of quantitative data suggested that HTPs (which included IQOS but also Eclipse, a more cigarette‑like HTP which uses a charcoal rod to heat tobacco, instead of electronic heating) were perceived as harmful, but less addictive and less harmful than cigarettes and more socially acceptable. Generally speaking, scores for perceived addictiveness, harmfulness, and social acceptability were similar regardless of smoking/e‑cigarette use status and on scale from 1 to 7 were approximately 6-6.5 for addictiveness, 6.5-7 for harmfulness, and 4-5.5 for social acceptability. Participants had lower use intentions for HTPs than for either cigarettes or e‑cigarettes; in terms of likelihood of next‑year use, this was the lowest for HTPs compared with cigarettes and e‑cigarettes among all participants combined and when assessed according to smoking/e‑cigarette use status among current smokers, e‑cigarette users, and dual users. The likelihood of use was negligible among non‑users of tobacco/nicotine products.

Two studies were described by Berg et al. [68,69] which arose from analyses of survey data from US‑representative and Israeli non‑representative samples of adults aged between 18 and 45. Participants were presented with IQOS advertisements and health warnings from the US market and asked to respond to questions regarding perceived health risks and intentions to try IQOS. In the first study [69], control (vs. reduced exposure) messaging resulted in significantly higher perceived relative harm compared with smoking (adjusted odds ratio (aOR): 1.29, i.e., approximately 30% higher), significantly higher perceived relative exposure (aOR: 1.34), significantly higher perceived relative disease risk (aOR: 1.23), and significantly lower likelihood of suggesting IQOS to smokers (aOR: 0.85). Reduced risk (vs. reduced exposure) messaging resulted in lower perceived relative harm (aOR: 0.86, i.e., approximately 15% lower). When the FDA‑authorised claim that ‘IQOS is a better choice for adult smokers’ was assessed, viewing this claim was associated with a ~20% greater likelihood of suggesting IQOS to smokers relative to control (aOR: 1.19). No interactions between risk/exposure messaging and messaging suggesting FDA endorsement were found. Additionally, Israeli participants, current cigarette smokers, and males perceived lower relative harm and exposure and greater likelihood of trying or suggesting IQOS to smokers. The second study [68] used a slightly different experimental design (4 x 3 factorial design compared with the 3 x 3 factorial design used in the first study) but reported substantially similar findings for risk perceptions, intentions to try, and the likelihood of recommending to smokers.

Risk Perception Studies in Asian Countries

Several risk perception studies have been performed in Asian countries in which HTPs have been launched. Using data from an online survey study which collected data from 2,300 male and 4,700 female Koreans aged between 20 and 69 in 2018, Kim et al. [70] investigated and assessed factors causing HTPs to be perceived as less harmful than cigarettes. Of the 7,000 participants, only 17% reported HTPs to be less harmful than cigarettes. Participants aged 20-34 years (aOR: 1.20), who were male (aOR: 1.26), and had an income above a certain threshold (aOR: 1.30) were more likely to perceive HTP use as less harmful than cigarette smoking, compared with those who were aged ≥ 50 years, female, and had an income below a certain threshold, respectively. Compared with exclusive smokers, never users of any of the three ‘tobacco products’ (cigarettes, HTPs, and e‑cigarettes) had lower odds of perceiving HTP use as less harmful than cigarette smoking (aOR: 0.64). However, exclusive HTP users (aOR: 3.32), dual users of HTPs and cigarettes (aOR: 2.25), dual users of HTPs and e‑cigarettes (aOR: 3.96), dual users of e‑cigarettes and cigarettes (aOR: 1.96), and triple users (aOR: 5.12) were more likely to perceive HTP use as less harmful than cigarette smoking. Regarding perceiving HTP use as being helpful to smoking cessation, this perception was less likely (aOR: 0.72) among those aged 35-49 but similar among those aged 20-34 and 50-69. Gender had no impact on this perception, but those with a low household income were 30‑40% less likely to perceive that HTPs can help smokers stop smoking than those with higher household incomes. Tobacco/nicotine use status also played a role in smoking cessation perceptions; relative to exclusive smokers, never users were less likely (aOR: 0.72) to perceive HTPs as helpful in smoking cessation, while exclusive and dual HTP use, along with other combinations of dual use and triple use, were strongly associated with a higher perception of cessation support. In a similar study from the same group, Kim et al. [71] found that, among 2,971 Korean adult tobacco/nicotine product users, current e‑cigarette or HTP users were more likely to perceive e‑cigarette or HTP use as being less harmful than cigarette smoking than non‑smokers/non‑users. Current tobacco/nicotine product users were also more likely to believe that HTPs or e‑cigarettes are acceptable to be used indoors, and this belief was associated with more frequent use of HTPs and e‑cigarettes at home or in a car. In a further analysis of data from a Korean nationally representative sample of 2,000 adults aged between 18 and 65, Park et al. [72] assessed perceptions of HTPs and intentions to quit among adult tobacco users. Mean values related to perceptions responses among exclusive HTP users in the domains of having a smell, producing smoke, leading to “secondhand” exposure, not being helpful to quit smoking, being unattractive, and being unaffordable, were all lower than mean values for exclusive smokers or exclusive e‑cigarette users. In a more recent South Korean study, Goulette et al. [73] assessed data from the 2020 ITC Korea survey, which included responses from adult smokers who were either exclusive smokers (n=1,845), dual users of HTPs and cigarettes (n=1,130), dual users of nicotine vaping products (e‑cigarettes) and cigarettes (n=224), and triple users of all three products (n=514). Among all respondents, only 27.6% believed that HTPs were less harmful than cigarettes, and 61.3% held the belief that HTPs were equally as harmful or more harmful than cigarettes. When data were examined based on tobacco/nicotine product use status, exclusive smokers were slightly more likely to believe that HTPs were equally as harmful or more harmful than cigarettes, and much less likely to believe that HTPs were less harmful, compared with respondents in the other three user groups. This led the authors to conclude that risk communication concerning HTPs should align with the best available scientific evidence [73], a suggestion which implies a need to correct misperceptions, particularly among smokers who would benefit from transitioning to HTP use.

Three studies have examined HTP risk perceptions in Japan. The first study used data from the 2018 ITC Japan survey, which collects cross‑sectional data in multiple waves over time from a nationally representative sample of Japanese cigarette smokers, HTP users, dual cigarette/HTP users, and non‑users regarding their knowledge, attitudes, beliefs, perceptions, behaviours, and use patterns associated with cigarette smoking and HTP use [74]. The aims of the study were to examine smokers’ harm perceptions of HTPs relative to cigarettes, differences in relative harm perceptions between exclusive smokers and smokers who use HTPs (dual users) and between dual users based on the frequency of product use, and if smokers who were exposed to HTP advertising held beliefs that are consistent with marketing messages of lower harmfulness [75]. Survey responses were obtained from 2,614 adult exclusive cigarette smokers and 986 dual users of cigarettes and HTPs. Almost half of smokers perceived that HTPs are less harmful than cigarettes, with approximately a quarter believing they are equally harmful and a quarter not knowing. Only a very small proportion of smokers (less than 2%) thought that HTPs were more harmful. Dual users were more likely than exclusive smokers to believe that HTPs are less harmful, and frequent HTP users were more likely than infrequent users to believe that HTPs are less harmful. HTP users were significantly more likely than non‑users to believe that HTPs are less harmful than cigarettes, and this belief was more prominent among frequent users. Believing that HTPs are less harmful than cigarettes was associated with noticing HTP advertising on television, in newspapers and magazines, on posters and billboards, in stores where tobacco or HTPs are sold, on social media, or in bars and pubs. This suggests that accurate advertising concerning the relative and absolute risks of HTP use may be useful in encouraging smokers to transition to using HTPs. In the second study, a similar belief, that transitioning to using novel THR products may be enhanced by providing accurate information and education, came from an analysis of cross‑sectional data from a Japanese Association of Smoking Control Science (JASCS) online survey of 277 physicians, pharmacists, nurses, and public health practitioners [76]. These data were used to assess healthcare provider knowledge of novel tobacco products and self‑efficacy to counsel patients about product use. More than half the sample had received previous training in treating tobacco use, but almost two‑thirds had no knowledge of HTPs. The accuracy of knowledge of HTP use risks was low. For example, only 14% of participants correctly stated that HTPs contain less harmful components than cigarettes. Knowledge of what was contained in HTP emissions was also low, with only a quarter of participants correctly stating that HTPs do not emit carbon monoxide. However, there was a much higher awareness (89% correct) that HTPs contain nicotine. Greater knowledge of HTPs was associated with male gender, higher rates of training at JASCS, and prior learning about HTPs at JASCS or via the internet. The third study assessed awareness, attitudes, and concerns about HTPs in a sample of physician members of the Japan Medical Association [77]. Among the 5,492 physicians surveyed, just over three‑quarters were aware of HTPs and approximately half had ever discussed using HTPs with their patients, though detail around what was discussed (e.g., stopping using HTPs, or asking smoker patients to switch to HTP use) was not disclosed. Regarding HTP perceptions, two‑thirds overall had concerns over the lack of evidence on the long‑term safety of HTPs, half reported an assumption that HTPs are less harmful than cigarettes, and almost half reported an assumption that HTPs do not have a passive impact on non‑users. Furthermore, approximately 40% had concerns regarding the long‑term impacts of nicotine addiction, though much smaller proportions thought that HTPs could perpetuate nicotine addiction or result in dual use (approximately 24% and 8%, respectively). A third also stated concern over a lack of regulatory oversight of HTPs [77]. Interestingly, perceptions of HTPs appeared more favourable among those physicians who were either ever or current users of HTPs, though this was not assessed statistically.

In a further Asian study, Wu et al. [78] assessed perceptions of HTPs using data from population‑based, cross‑sectional surveys among Cantonese, Chinese‑speaking participants in Hong Kong aged 15 and above in 2018 and 2019. Just over one‑quarter of participants perceived that HTP use is less harmful than cigarette smoking, and 19% believed that HTPs are effective in helping smokers stop smoking. In a further analysis of support for regulations governing HTPs, participants who perceived HTPs as less harmful than smoking or as effective stop-smoking aids were less likely to support bans on promotion and advertisements, bans on use in smoke‑free areas, and a total ban on HTP sales.

Risk Perception Studies in European Countries

Eight studies have assessed HTP perceptions and beliefs in European countries, namely, Poland, Hungary, Romania, Italy, Spain, and the UK. In a survey study of 1,344 medical students in Poland conducted in 2019, Majek et al. [79] assessed awareness and opinions about HTPs, alongside the prevalence of HTP use and smoking habits. HTP use prevalence was found to be low in this population, with only 1.1% of females and 2% of males reporting exclusive use of HTPs and a similar proportion reporting HTP/cigarette dual use. Among those who were aware of HTPs (those unaware were excluded from analyses), only a small proportion (5.3%) perceived HTPs as being 'safe for your health', though this proportion was larger (43%) among those who were HTP users. More than 90% of participants, regardless of HTP use status, perceived HTPs as a possible addiction risk. HTP non‑users were much more likely to perceive HTPs as being as addictive as cigarettes, and HTP users were more likely to respond that HTPs are less addictive. Similarly, HTP non‑users were much more likely to perceive HTPs as posing a passive smoking risk and not being safe for use by pregnant women, and HTP users were more likely to respond that HTPs do not pose such a risk and are safe for that user group. In a much broader nationally representative sample of Polish individuals aged 15 and above in 2019, Jankowski et al. [80] also assessed perceptions of the harmfulness of HTPs and other tobacco or nicotine products, including smokeless tobacco, shisha, e‑cigarettes, cigarettes, and cigarillos. Most participants were cognisant of the health risks associated with cigarette smoking, and the vast majority of participants (72%) also believed that HTPs were as harmful as cigarette smoking, with 22% believing HTPs were less harmful and 6% believing they were more harmful. In a further Polish study, which was confined to assessing the beliefs and perceptions of 12,000 Polish adolescents aged between 13 and 18, Wężyk‑Caba et al. [81] found that perceptions of risk were much lower than those described in the more general, older population [80]. Thus, 62% of the adolescents surveyed perceived HTP use as being less or far less harmful than cigarette smoking [81]. The likelihood of perceiving HTP use as less harmful than smoking was associated with being male, with having ever used HTPs, and with being exposed to tobacco advertising.

In a Hungarian study, conducted among 1,423 ever or current adult HTP users who participated in a cross‑sectional web‑based survey in 2020 [82], 86% of participants believed HTP use to be less harmful than cigarette smoking, and current HTP users were more likely to support this belief. Perceptions of reduced risk were associated with being male, being educated to a higher level, and living in a major city as opposed to a town or village. In Romania, young adults aged between 18 and 26 took part in qualitative study interviews to assess the perceptions of using HTPs following exposure to direct marketing methods [83]. The thematic analysis used in the study identified three major themes concerning HTP use: (1) people, places, and subjects of marketing, and that they consider themselves above the influences of marketing; (2) engagement with risk narratives that may influence their decision to use HTPs;and (3) and social body, family bonds, and autonomous self. Young adults’ decisions to use HTPs were further suggested to be enhanced by the attractiveness of the product (novelty, inviting appearance, technological appeal, and price) and a presumed less damaging impact on health. In another qualitative study conducted on pregnant women, a population particularly susceptible to the health impacts of tobacco use [84] in Italy, Maglia et al. [85] assessed perceptions, thoughts, experiences, and feelings about HTPs, as well as cigarettes and e‑cigarettes. Participants who were using HTPs generally expressed certainty that they pose less risk than cigarettes and expressed unanimous mistrust of information regarding the potential risk of HTP use to their unborn babies; nevertheless, most of them decided to quit HTP use during pregnancy.

Similar to the Japanese study of physicians’ perceptions [77], Jimenez Ruiz et al. [86] assessed the prevalence of use, knowledge, and perceptions among a representative sample of members of the Spanish Society of Pneumonology and Thoracic Surgery (SEPAR) in 2022. Analogous to the Japanese study, approximately 70% knew about IQOS, and 60% considered IQOS to not be safe or effective for smoking cessation. However, 11% of SEPAR members agreed that IQOS use can minimise the harms associated with tobacco use [86].

In a cross‑sectional survey study conducted in London, UK, Kale et al. [87] examined identity (e.g., asking smokers whether they thought that ‘smoking is a part of me’), attitudes, and indicators of effectiveness, among small groups of both smokers and ex‑smokers who had been using HTPs for more than three months. Smokers and HTP users were found to have a similar strength of identity and had similar scores on the Mood and Physical Symptoms Scale [88] in the domains of both product‑specific craving and mood and physical symptoms. However, HTP users had significantly lower cigarette‑specific cravings and significantly higher intentions to stop using the product they were using, than smokers. In addition, HTP users reported a strong attitude towards recommending HTPs to a friend who wants to stop smoking (mean value of 4.4 on a scale from 1 to 5) and believing that HTPs were efficacious in smoking cessation (4.5). Attitude scores for addictiveness and safety (both compared to smoking) were much lower, at 2.6 and 2.1, respectively [87].

Other Risk Perception Studies

Four further studies were identified by the literature search. Morgan et al. [89] assessed harm perceptions and beliefs about potential MRTPs, including HTPs, among 864 adult current and former smokers in the US in 2019. After reading descriptions of MRTPs, and a description of HTPs, e‑cigarettes, and snus, participants answered questions about awareness, harm perceptions, use, the likelihood of use, beliefs, and intentions to use the three types of products that have the potential for, or have already received, modified risk claim authorisation from the US FDA. Awareness of HTPs was only reported by approximately 12% of participants, and very few participants (less than 1%) had ever used them. Mean perceptions of harm on a scale from 1 to 4 (with 4 meaning highest harm) were 2.6 for HTPs, 2.7 for e‑cigarettes, and 2.8 for snus. The likelihood of use, on the same scale, was lowest for snus (mean: 1.5), higher for HTPs (1.8), and the highest for e‑cigarettes (2.1). The three most endorsed beliefs about using HTPs were that they contain nicotine, that their use is risky, and that they cause lung damage. Participants were least likely to endorse the beliefs that HTPs would help smokers quit, that it was not addictive, and that it was cool. Relative to cigarettes, participants most frequently endorsed the belief that HTPs are expensive, that the science about HTPs is untrustworthy, and that HTPs are appealing to kids (not defined). Similar to their beliefs about using HTPs, participants were least likely to endorse the belief that HTP use had long‑term benefits and was not as addictive as smoking cigarettes. Regarding intentions to use HTPs, beliefs that heated tobacco would taste good and would be a good quitting aid were associated with increased odds of intentions to try HTPs while believing that HTPs are expensive was associated with lower odds. In a similar study among 424 US and Israeli adults who were aware of HTPs and reported current use of cigarettes, e‑cigarettes, HTPs, hookah, cigars, pipe, or smokeless tobacco [90], almost 90% of participants had noticed HTP health warning labels and among these, approximately one‑quarter each were either concerned or reassured about HTP use, and approximately half reported no effect. Factors associated with increased concerns about HTP use were using other tobacco products (i.e., not currently using HTPs), and being female. Factors associated with being reassured about HTP use included being a current HTP user, being from Israel, being less educated, and being female. Reporting that HTP health warning labels increased concern or reassured of use were positively associated with HTP use intentions; no associations with risk perceptions were found [90].

In a study among 2,038 Guatemalan adolescents aged between 13 and 18, Monzón et al. [91] used a discrete choice methodology to assess the effects of product type and characteristics on appeal and perceived harm of various tobacco/nicotine products including HTPs, although assessment is impeded somewhat by the inclusion of an e‑cigarette as a HTP due to the availability of only a single HTP at the time in Guatemala. Product type accounted for almost 90% of the variation in participants’ choices. Participants were less interested in trying HTPs and viewed them as more harmful compared to cigarettes. Conversely, they were more interested in trying e‑cigarettes which were perceived as less harmful compared to cigarettes.

Summary of HTP Risk Perception Studies

In summary, many studies have described misperceptions of the risk of HTPs relative to cigarette smoking, with widespread belief that HTP use is equally or more harmful than smoking. Risk perceptions differed in many studies depending on the tobacco use status, with HTP and e‑cigarette users, for example, more likely to have perceptions of lower risk than cigarette smokers. Non‑smokers also appeared more likely to have perceptions of increased risk, and this could help to minimise HTP use among nicotine and tobacco non‑users. Between‑country and potentially cross‑cultural differences were also observed, as well as differences due to demographics such as age, education level, and income. Some studies highlighted the drivers of risk perceptions. Exposure to messaging, advertising, and health warnings impacted risk perceptions, though this was not unexpected. One interesting driver of reduced risk perceptions was differences in the physical aspects of HTP use compared with cigarette smoking, for example, heating and not burning tobacco, whereas similarities between HTP use and cigarette smoking, such as the same ritualistic experience and the use of sticks that resemble cigarettes, were drivers of increased risk perceptions. Overall, while the higher perception of risk among nicotine and tobacco non‑users may be beneficial from a population‑level THR perspective since it may help to limit uptake in this population, perceptions of equal or higher risk among smokers could be detrimental to THR since they could prevent uptake of the potentially less harmful HTP. This may suggest the need for better communication of the relative risks associated with HTP use and targeting this communication towards the intended audience, i.e., adult cigarette smokers, while avoiding reducing risk perceptions and promoting initiation among unintended users. In addition, since some studies noticed changes in perceptions over time, this suggests that perceptions of HTPs should be frequently monitored to assess future changes and their potential impact on THR.

Unintended Use and Awareness Among Youth and Young Adults 

The use of non‑combustible tobacco and nicotine products, including HTPs, by youth may serve to undermine their harm reduction potential, particularly if the use of a novel tobacco/nicotine product acts as a ‘gateway’ into cigarette smoking [48,92]. For this reason, generating an estimate of youth HTP use, and any potential future behavioural impact of that use, such as smoking initiation, is critical to a full understanding of HTP harm reduction potential.

Asian Studies on Youth and Young Adult Awareness and Use 

In Korea, a country in which HTPs were marketed before many other countries, Lee et al. [93] used data from the nationally representative 2018 Korea Youth Risk Behavior Survey (KYRBS) to examine the use of HTPs among adolescents and explore any associations with the perceived stress, physical activity, and internet use, noting that data collection took place approximately one year following the introduction of HTPs into South Korea. Among the 60,040 participants, who were aged between 12 and 18, HTP ever-use prevalence was very low. Fifty‑nine participants (0.1% of the survey population) reported exclusive ever HTP use, 147 (0.3%) reported dual use of HTPs and cigarettes, 92 (0.2%) reported dual use of HTPs and e‑cigarettes, and 1,217 (2.3%) reported triple (HTP, cigarette, and e‑cigarette) use. Ever-use exclusively of cigarettes was reported among 7.9% of the study population. The proportion of participants in any of the HTP ever-use categories was higher in older (high school) compared to younger (middle school) participants, and triple use (but not exclusive HTP use) was generally higher in those performing poorly academically, among those of low family economic status, and among those who also reported drinking alcohol. In general, HTP use was associated with having low perceptions of personal stress, although triple use was significantly more common among those who reported high perceived stress and was associated with being more physically active and not reporting internet use. Also arising from the analysis of the 2018 KRYBS survey, Kang et al. [94] reported a similarly low prevalence (2.8%) of ever use of HTPs. Of those who had ever used HTPs, 9% were never smokers, 91% were ever smokers, and three‑quarters currently smoked. HTP use was also strongly associated with ever use of e‑cigarettes. The study also examined smoking quit attempts, finding that the likelihood of having made a quit attempt was similar among HTP users and non‑users. The findings of both Lee et al. [93] and Kang et al. [94] were corroborated in a further study examining 2019 KRYBS data [95], which found that HTP use prevalence was low overall, that triple product use was most common, and that HTP use was strongly associated with both cigarette and e‑cigarette use. Similar findings of a very low prevalence of ever use of HTPs among the total population and a much higher prevalence of triple product use were reported by Kang et al. [96], although this is perhaps unsurprising since the paper analysed the same 2018 KRYBS dataset as that of Lee et al. [93]. Similarly, HTP use was strongly associated with self‑reported risky alcohol drinking [96], a factor which, along with sexual experience, drug use, depression, and living in a metropolitan area, was associated with poly use of tobacco and nicotine products (HTPs, cigarettes, and e‑cigarettes) in an analysis of 2019 KRYBS data [97].

Han [98] used data from the 2019 KRYBS survey to assess a different factor associated with HTP use, that of the ability to purchase tobacco products, including HTPs, among Korean youth. Across the whole survey population, the majority of participants (93%) had not tried to purchase tobacco and nicotine products. Among those that had, the proportion of those who found it ‘impossible’ to purchase tobacco and nicotine products was similar to the proportion who were able to do so ‘without any effort’. The prevalence of HTP use was lower than that of both cigarettes and e‑cigarettes, and perhaps unsurprisingly the prevalence of use was lower among those who found buying tobacco and nicotine products difficult.

Adolescent and young adult use of HTPs has also been examined in Japan, another market in which HTPs were launched before other markets. Okawa et al. [99] analysed survey responses from participants aged between 15 and 29 using data from the 2019 Japan New Society and New Tobacco Internet Survey (JASTIS) study, a longitudinal internet prospective cohort study which investigates perceptions, attitudes, and use of heat‑not‑burn tobacco (i.e., HTPs), e‑cigarettes, and conventional tobacco products in Japan [100]. Similar to the Korean studies described above [93-96], HTP use was strongly associated with the co‑use of other tobacco/nicotine products, including cigarettes and e‑cigarettes [99]. Using data from a different Japanese nationally representative adolescent survey, the 2017 Lifestyle Survey of Adolescents, Kuwabara et al. assessed the use of HTPs, e‑cigarettes, and cigarettes, among 64,152 students aged between 12 and 18 [101,102]. Similar to the Korean studies, the prevalence of HTP current use and ever use was very low (less than 1% for current use and less than 3% for ever use). Both current and ever-use likelihoods increased with education grade level, a finding similar to that reported in one of the Korean studies [95]. Additionally, HTP current and ever use were lower than the use prevalence of both cigarettes and e‑cigarettes [101,102], a finding similar to that seen in some of the Korean studies [93-95,97].

Igarashi et al. [103] assessed data from a cross‑sectional online survey study conducted across Japan in 2017 and explored sociodemographic variables associated with tobacco product use among young adults and adults. HTP use, which included both exclusive use and co‑use with other tobacco products, was the highest among the youngest age cohort surveyed (aged between 20 and 29). HTP use prevalence decreased with increasing age and increased with income level among both men and women. Regarding occupation status, those with “Part‑timer” and “Inoccupation” were significantly less likely to be HTP users than those with “Clerical” status among men. Regarding education status, those with “University and Graduate school” were significantly more likely to use HTPs than those with “Junior high school and High school” among women. Overall, the study appears to suggest that HTP use is focussed on individuals who are younger and have a higher income level.

Two studies have examined HTP use among adolescents in Taiwan. It is notable that at the time at which both studies were conducted, HTPs were not legally available for sale in Taiwan. In one of the studies, Lee et al. assessed data from the 2018 Taiwan Global Youth Tobacco Survey to identify the risk factors for cigarette, e‑cigarette, and IQOS use among 49,971 adolescents in school grades 7-12 (approximate ages: 12-18) in Taiwan, focussing particularly on socioeconomic status, smoking status of parents and peers, cigarette promotions, and anti‑tobacco campaigns [104]. Regarding current IQOS use, this was higher among boys than girls, it generally increased as age increased, it was generally higher among those with a higher monthly allowance, and it was generally lower if the participants’ parents had a higher (postgraduate‑level) education level. Some other interesting factors were highlighted in these analyses. IQOS use among an estimated population of 35,011 current users (2.3% of the adolescent population) was least likely (5% of IQOS users) if only the mother smoked but was seen in 33% of participants whose fathers smoked and in 44% of those where neither parent smoked. IQOS use was also significantly higher if close friends smoked, significantly lower if participants had access to free cigarettes compared to if they did not, and was significantly lower among those exposed to cigarette advertisements compared to those who were not [104]. In the second study, Chang et al. [105] used data from the same survey to assess IQOS use prevalence, finding that approximately 2% of Taiwanese adolescents were current IQOS users and that approximately 4% had tried using IQOS.

One other study examined HTP awareness and use among youth and adolescents in China [106] following a retrospective analysis of data from the Hong Kong Youth Quitline Cohort of youth smokers, a survey which has collected data on HTP use among youth smokers aged 14-25 since early 2017. The survey data suggest that the number of youth using HTPs has grown since 2017 in Hong Kong, although the absolute prevalence is low and a plateau does appear to have been reached by 2020. HTP users tended to be male, single, of higher educational status, students, and part‑time employed, and almost 90% of HTP users had a previous history of use of other tobacco products. The study also assessed perceptions of HTPs; participants were generally aware that HTPs are addictive and that HTPs contain less harmful substances than cigarettes. However, participants generally disagreed that HTPs can help to stop smoking or that they can reduce cigarette consumption. In this regard, the use of HTPs as a smoking cessation aid was ranked the lowest among reasons for using HTPs, and curiosity and peer influence were ranked the highest.

European Studies on Youth and Young Adult Awareness and Use

A small number of studies have assessed youth HTP use in European countries. Havermans et al. examined awareness, perceptions, and use of, cigarillos, HTPs, and tobacco-free nicotine pouches among Dutch adolescents, as well as among Dutch adults [107]. Awareness of HTPs was found among 20% of those aged between 13 and 17, and 29% of those aged between 18 and 24. These values were not dissimilar to those seen among older adults, i.e., those aged 25 and older. Youth/young adult awareness was higher among those who had smoked more than 100 cigarettes in their lifetime and current smokers. Ever use of HTPs was reported in less than 1% of those aged between 13 and 17 and in approximately 4% of those aged between 18 and 24. Current HTP use was not found among those aged 13-17 and was reported in 0.5% of those aged 18-24. Both ever and current HTP use were higher among ever and current smokers, and there was a degree of poly use of HTPs with other tobacco and nicotine products such as oral tobacco, nasal tobacco, tobacco-free nicotine pouches, and tobacco snus. A study in Denmark also assessed youth use of HTPs using a baseline data sample from a longitudinal study assessing tobacco product use among Danish youth and young adults aged between 15 and 29 [108]. Similar to the Dutch study [107], the prevalence of ever use of HTPs was very low and was found in only 3% of the survey population [108]. Ever use was more common in males and the highest among those aged between 25 and 29. Occasional HTP use was extremely low (0.2%), while daily use was almost non‑existent (0.1%). Of interest, the data also show that cigarettes were the most common tobacco product used first, and no subjects used HTPs before using other tobacco products. In a Swiss study assessing the use of tobacco, nicotine, and cannabis, products among students aged between 15 and 21 in Switzerland [109], similar findings to those observed in Holland [107] and Denmark [108] were found in that the use of HTPs was very low and that daily use of HTPs was almost zero. Notably, the use of HTPs was lower than that of any of the other tobacco products assessed, including cigarettes, snus, snuff, hookah, pipes, cigars, and cigarillos [109]. In a study assessing the use of tobacco products among Croatian university students aged 18 and over [110], no data were presented regarding the overall prevalence of the use of HTPs. However, their use was seen among 18% of current users of any tobacco product, which was significantly lower than that for cigarettes (75%) but higher than that for e‑cigarettes (8%). In a similar study conducted among Milanese university students [111], the majority of students were reported to be non‑smokers (never smokers and former smokers combined). Approximately two‑fifths of students were current cigarette smokers, and only 4% were current HTP users, a value similar to the proportion of current e‑cigarette users, and slightly higher numbers of students reported ever use of HTPs or e‑cigarettes. Dual use of cigarettes and e‑cigarettes/HTPs was also low, with a prevalence of such use of approximately 4% among the student population.

Youth and Young Adult Awareness and Use in the United States

Many studies have examined awareness of, interest in using, and actual use of, HTPs in the US, although it should be noted that HTPs have not been made widely available in this country subsequent to the Premarket Tobacco Product Application (PMTA) marketing order for IQOS in 2019. Using data from Wave 1 of the ITC Youth Tobacco and E‑Cigarette Survey collected in 2017, Czoli et al. assessed awareness, interest in trying, and susceptibility to trying, IQOS among 12,064 youth aged between 16 and 19 who were shown images of IQOS prior to responding to online survey questions regarding awareness of, and interest in trying, IQOS [112]. Data were collected from youth in three countries: the US, Canada, and England. Awareness was the highest among US youth, with 9% of participants aware of the product, compared with approximately 6% in both Canada and England. This is potentially due to media interest following the submission of the IQOS PMTA in the US earlier in the same year that the survey was conducted. When examining susceptibility among those with different tobacco/nicotine product use statuses, in all countries, interest in trying IQOS and susceptibility to its use was the highest among current smokers, lowest among never smokers, and intermediate among both former and experimental smokers. Similarly, prior or current e‑cigarette use status was also associated with both interest and susceptibility to trying IQOS. Overall, susceptibility to trying IQOS (25%) was higher than for cigarettes (19%), but lower than for e‑cigarettes (29%) among youth never smokers and never e-cigarette users across all three countries. When the cross‑sectional ITC data collection described by Czoli et al. [112] was extended over time, from 2017 through 2019, the actual use of IQOS was found to be negligible across the three countries, with a use prevalence of approximately 1%. The prevalence of use of other products, including cigarettes, e‑cigarettes, hookah, smokeless tobacco, and NRT, was generally higher than the prevalence of HTP use. In a similar study to that of Czoli et al. [112] conducted in 2018 among 332 young adults aged between 18 and 30, Phan et al. [113] reported similar findings in that curiosity, interest, and likelihood of using IQOS were the lowest among non‑tobacco users, higher among exclusive e‑cigarette users, and the highest among both exclusive smokers and cigarette/e‑cigarette dual users. Additionally, similar to Czoli et al. [112], males appeared more susceptible to IQOS use than females [113].

Data from the National Youth Tobacco Survey (NYTS) have been used in various assessments of youth use of HTPs in the US. Note, however, that the wording of the NYTS questions regarding HTP use in all versions of the survey to date gives rise to the possibility that respondents were answering questions related to carbon‑heated (e.g., Eclipse) as well as electronically heated (e.g., IQOS and glo) HTP use. When assessing the 2019 NYTS data, Dai et al. [114] reported that awareness, ever use, and current use of HTPs was found among 12.8%, 2.4%, and 1.6%, of US youth, respectively. For each of those use statuses, greater proportions of participants were male, Hispanic, current tobacco users, current smokers, or e‑cigarette users and were exposed to HTP use in their household. Similar findings were reported by Gentzke et al. [115] following their analyses of the 2020 NYTS data, with an overall past 30‑day use prevalence of HTPs of 1.3% reported. Use prevalence was lower than that seen for cigarettes, cigars, e‑cigarettes, and smokeless tobacco and was higher among Hispanic youth. By 2021, the prevalence of past 30‑day HTP use had fallen to 0.7% [116], although it should be noted that there are methodological differences in the data collection procedures between the 2020 (questionnaire completed in the classroom) and 2021 (online survey completed in the classroom or at home) NYTS surveys, which may hinder direct comparisons across different survey years.

In a slightly more complex analysis of the 2019 NYTS data, Lee et al. [117] also reported higher awareness and use of HTPs among Hispanics, among those with familial HTP use, and among those with past 30‑day use of flavoured, non‑cigarette tobacco product use. In addition, both ever and current use of HTPs were strongly associated with the use of one or more other tobacco products, and particularly with poly use of two or more other products. Casseus et al. [118] also used the 2019 NYTS data to examine the use of HTPs among youth with a cognitive disability, determined by self‑reporting of a physical, mental, or emotional condition that gives rise to serious difficulty concentrating, remembering, or making decisions. Those with cognitive disability were approximately twice as likely either to have ever used, or to currently use, HTPs, noting that there was also a co‑association with being of Hispanic ethnicity.

In the most recent examination of NYTS data, Birdsey et al. [119] reported a very low (1.0%) prevalence of past 30‑day HTP use among US middle and high school students in 2023, which approximates an overall estimated weighted number of students of 260,000. This prevalence estimate was lower than for almost all other tobacco/nicotine products assessed, including cigarettes, e‑cigarettes, cigars, smokeless tobacco including tobacco‑free nicotine pouches, and hookah. Of further note, the HTP use prevalence estimate equates to only 10% of all US youth tobacco/nicotine product use in 2023 [119].

Using data from a single wave of the longitudinal CHOICE‑STRATA cohort study, which collects data from a representative sample of students in middle schools in Southern California, Dunbar et al. [120] examined awareness and use of HTPs among adolescents aged between 11 and 13 in 2018 and 2019. Awareness and use of HTPs were significantly associated with being male and were associated with being lesbian, gay, or bisexual, although this effect did not reach statistical significance, and generally speaking awareness and use were higher among non‑Hispanic White students. In addition, awareness and use were significantly associated with a history of use of many other types of tobacco and nicotine products, with higher cigarette consumption and cigarette dependence scores, as well as with a history of marijuana and other drug use. Another Californian study collected data from a school‑based survey of tobacco use among 150,516 middle and high school students in 2019 and 2020 [121]. Overall awareness of HTP use was seen among 9% of students, and the prevalence of ever use of HTPs was less than 1%. The current use prevalence of HTPs was extremely low and was found in only 0.2% of the survey population. Ever and current use of HTPs were associated with having ever smoked, but not with having ever used e‑cigarettes.

Duan et al. [122] used data from a survey study conducted in 2020 in six major metropolitan areas in the US to assess awareness, use, and use intentions of HTPs among 2,470 young adults aged between 18 and 30. Notably, one of the metropolitan areas, Atlanta, Georgia, was a launch city for IQOS in 2019. Awareness of HTPs was the highest in Atlanta and was reported by approximately 4% of participants. While the prevalence of ever use of HTPs was among the highest in Atlanta at 4%, Seattle had the highest prevalence of ever use (6%). HTP ever use across all metropolitan areas in the study was reported by 101 of the 2,470 participants, which is approximately 4%. Both awareness and ever use were more common among males compared to females, were most common among White participants and the lowest among Black participants, and were strongly associated with past 30‑day use of either cigarettes or e‑cigarettes, or other tobacco products. Past 30‑day use of other tobacco products was also strongly associated with ever use of HTPs, although this was based on data for little cigars/cigarillos, hookah, and smokeless tobacco, collapsed into a single ‘other tobacco product’ category. For past 30‑day users of each individual product type, however, the prevalence of ever use of HTPs was low, particularly for smokeless tobacco. Intention to use HTPs was generally very low and reported by approximately 1% of participants although it was, perhaps unsurprisingly, slightly higher (3%) in those who had ever used HTPs. 

Using data from the 2020‑2021 Cannabis, Obesity, Mental health, Physical activity, Alcohol, Smoking, and Sedentary behaviour (COMPASS) study, Mott et al. [123] examined whether there was an association between proximity to, and density around, schools of retailers selling IQOS and youth use of HTPs. Only a small number (10%) of schools were found to have an IQOS device retailer within 1 km of schools, though the number of retailers selling IQOS tobacco consumables (HEETS) in this proximity to schools was much higher (65%). When estimating the likelihood of current HTP use among students, less than a quarter of the variability in the likelihood of use was accounted for by retailer proximity [123]. In addition, the study identified an HTP use prevalence of less than 1% among students, a figure similar to that reported following NYTS data analyses described above. While the authors’ assertion that continued monitoring of HTP use is warranted is appropriate, their further assertion that the findings suggest that students may be acquiring HTPs through other, non‑retail sources was not supported by any evidence.

One final US study identified came from an analysis of data from the Altria Client Services Underage Tobacco Use Survey, which collected data from nationally representative samples of youth and young adults aged 13-20 between 2020 and 2022 [124]. Awareness of HTPs and snus were similar and the lowest of all the tobacco products assessed, reported by approximately 15‑20% of participants aged 13-17 and 20‑30% of those aged 18‑20. In contrast, awareness of cigarettes and e-cigarettes was close to 100%. These awareness levels were generally static over time. Ever use of HTPs was categorised as low to very low, reported by approximately 1‑2% of those aged 13‑17 and 2‑4% of those aged 18‑20. Past 30‑day use of HTPs was generally negligible, reported by less than 1% of participants regardless of age group.

Other Studies on Youth and Young Adult Awareness and Use

Outside of Asia, Europe, and the US, an additional study examined HTP use among youth and young adults in another country in which HTPs are marketed, Guatemala. Gottschlich et al. [125] used cross‑sectional data obtained from a survey study conducted in Guatemala City assessing awareness, susceptibility, and use of HTPs among youth aged between 13 and 17. Among those surveyed, half were aware of, and susceptible to, HTPs. Ever use was observed among 8% of the population, while current use was seen in 3%. HTP use before the use of other tobacco/nicotine products was low, observed among less than 2% of ever users; cigarettes (43%) and e‑cigarettes (54%) were more commonly used. Among those who reported using HTP, dual use was common, most frequently with e‑cigarettes (93%) but also with cigarettes (52%).

Summary of Youth and Young Adult Awareness and Use

Overall, regarding studies assessing youth/adolescent and young adult use of HTPs, the prevalence of use in the many regions and countries assessed was low to very low, regardless of whether ever/experimental use or established/frequent use was assessed. In many studies, the prevalence of use of HTPs was much lower than that of other tobacco and nicotine products, including cigarettes, e‑cigarettes, and oral nicotine products (e.g., tobacco-free nicotine pouches), and some studies reported an extremely low level of HTP use among never-smoking youth and young adults. HTP use also tended to be associated with being male, and in some US studies, HTP use was found to be more common among the young Hispanic population, though this finding was not common across all studies. One common finding across multiple studies was that HTP use was associated with the co‑use of one or more other tobacco and nicotine products. Whether there is a causal link cannot easily be determined from cross‑sectional studies, and potentially this poly‑use pattern arises due to common liability, a theory that suggests that the propensity to try tobacco products influences the patterns of use [126]. While the Guatemalan and Danish studies by Gottschlich et al. [125] and Bast et al. [108], respectively, support the common liability theory and refute the ‘gateway’ theory, longitudinal studies are required to better assess whether HTP use is common among young never users of tobacco and nicotine and whether such use leads to smoking initiation. In addition to longitudinal studies, survey research among young people may benefit from some degree of standardisation regarding terminology. It is apparent from the numerous studies assessed that different definitions of youth, adolescents, and young adults are used by different research groups. Standardising the terminologies would allow for better between-study comparisons to be made, as well as facilitating a better understanding of HTP use among young people.

Adult Awareness and Use

Many studies have examined awareness and use of HTPs among adults in the markets in which they are available globally, assessing adult HTP use in a number of regions and countries.

Adult HTP Use in Asian and Middle Eastern Countries

Japan is the fifth-largest cigarette market in the world. It was the first country in which HTPs were launched in 2014 and is the largest global market for HTPs [127]. Given this, several studies have examined data from the Japanese JASTIS survey to assess HTP use prevalence. Data from the JASTIS surveys are particularly useful since the survey is conducted annually, facilitating an examination of market trends and dynamics over time. The first study examined interest in HTPs and the prevalence of use by assessing the study follow‑up data in 2015, 2016, and 2017 [128]. The study also assessed Google Search records for HTP‑associated words, including several HTP brand names, which unsurprisingly increased after the launches of the various HTPs in Japan. Regarding the prevalence of use, 0.3% of respondents were current users of IQOS, and the same proportion were current users of Ploom TECH, a hybrid HTP which generates tobacco vapour by heating a liquid within a cartridge and passing it through a tobacco capsule. In 2017, these had risen to 3.6% for IQOS and 1.2% for Ploom TECH, and the prevalence of use of the glo HTP was 0.8%. The prevalence of use was higher among males, and smokers who had expressed an intention to quit smoking were more likely to be IQOS users compared to those expressing no intention to quit. In addition, logistic regression models identified intending to quit as a highly significant predictor of IQOS use. Compared with never smokers, current smokers were 11 to 19 times more likely to be current IQOS users, while the likelihood of former smokers using IQOS was no different from never smokers. In a cross‑sectional analysis of data from the 2017 JASTIS survey, Sugiyama et al. [129] assessed exclusive and poly use of tobacco products, including HTPs. At this time, exclusive use of HTPs was found among 5% of the population, while poly use including HTPs was found among 11%. Poly‑use of tobacco products was higher among males and younger adults aged 25-34 and was more likely among those with lower perceptions of risks associated with e-cigarette/HTP use, though these statistics were not reported according to which individual product types were being concurrently used.

The findings of Tabuchi et al. [128] described a rapid rise in HTP use between 2015 and 2017. This was explored further by Hori et al. [130], who estimated the prevalence of HTP use between 2015 and 2019 using JASTIS data. HTP use in Japan increased from 0.2% in 2015 to 11.3% in 2019. In that later year, Ploom TECH was the predominant HTP being used, with IQOS use slightly lower and glo use lowest of all. In contrast to the findings of Tabuchi et al. [128], Hori et al. [130] reported that the use was lower among those smokers with an intention to quit than those without such intention. The difference in results between studies is difficult to reconcile, though the authors attribute this to the changing profile of HTP users over time. Extending this analysis of changes in HTP use in Japan over time, Odani et al. [131] assessed 2020 JASTIS data, finding that the prevalence of current HTP use that year was 11%. This suggests that HTP use has plateaued, despite the introduction of further HTPs into the Japanese market. The analysis of 2020 data also found no difference in HTP use among smokers with and without intentions to quit smoking, which corroborates the suggestion by Hori et al. [130] of changing HTP use dynamics over time. The authors also suggested that HTP use was a ‘gateway’ to the use of other products, including cigarettes and e‑cigarettes [131]. Although the prevalence of HTP use was higher among users of those other products, it is not possible from this type of cross‑sectional methodology to elucidate if the use of one product results in the subsequent use of another. An alternative explanation could be the common liability principle [126]. Further extending the analyses of JASTIS data over time, Odani et al. [132] assessed 2022 data. They reported a prevalence of HTP use at approximately 12%, the majority of which was found among males. This supports the previous suggestion by Odani et al. [131] that HTP use had plateaued in Japan by 2022. Although not specifically analysed in any of the JASTIS studies, it is interesting to note that the prevalence of cigarette smoking had fallen in Japan from approximately 22% in 2015 [128] to 19% in 2022 [132]. While this reduction in smoking prevalence may appear to be modest, it may be indicative of a trend associated with the rise in HTP use, and this may be further indicative of HTPs displacing cigarettes and contributing to THR.

Other studies have used data from JASTIS to not only monitor changes in HTP use prevalence over time but also to assess longitudinal transitions in tobacco product use behaviour within individuals. Similar to Tabuchi et al. [128], Hori et al. [133] reported a rapid elevation in HTP use prevalence between 2015 and 2018 and reported that this increase was more common in older adults, particularly those aged between 40 and 59. Despite the group’s previous assertions of a ‘gateway’ effect of HTPs [131], the transition from HTP use to cigarette smoking among never smokers was not assessed and data from Hori et al. [133] showed that HTP use among never smokers was extremely low, at less than 0.5%. The study also indicated that former smokers were three to seven times more likely to use HTPs than never smokers and that current smokers were 7.5-60 times more likely [133].

Data from JASTIS have also been used to examine associations between time‑based smoke‑free policies in the workplace restricting tobacco product use [134]. Studies report that the prevalence of HTP use was lower among individuals working in workplaces in which tobacco product use was banned at lunchtimes compared with those working where there was either no such lunchtime ban or no time‑based tobacco product use policy. JASTIS data have also been analysed to assess HTP use among individuals with chronic diseases, such as diabetes, cardiovascular disease, chronic obstructive pulmonary disease (COPD), and cancer [135]. Studies report that people with these chronic diseases are more likely to use HTPs, either exclusively or dual‑using with cigarettes, than those without these diseases. A similar finding was reported by Kioi et al. [136]. These findings may not be surprising given that the majority of HTP users are either current or former smokers. It could also indicate a desire among those with chronic disease which may be associated with cigarette smoking to use HTPs as a means of either reducing or stopping cigarette consumption to minimise symptom exacerbation and further disease progression/harm.

JASTIS data have also been used to examine HTP use among Japanese middle‑aged and older adults with COPD, asthma, and overlapping asthma‑COPD (ACO) [137]. The proportions of HTP use were 3.0%, 0.6%, and 0.0% in never smokers; 7.8%, 13.2%, and 4.4% in former smokers; and 50.6%, 16.8%, and 68.0% in those with COPD, asthma, and ACO, respectively. After adjusting for confounders, aORs for HTP use in current smokers were 2.88 for COPD, 1.23 for asthma, and 5.81 for ACO compared to those without each disease [137]. While the authors suggest a causal link, it is important to consider that the association between HTP use and lung disease may not be causal. For example, it is possible that this is an example of reverse causation in that those smokers who developed lung disease were more likely to initiate HTP use as a means of preventing further harm. 

Some papers have described assessments of data from the ITC Japan survey, a study similar to JASTIS, to estimate the prevalence of HTP use. The earliest study examining data from this source assessed the prevalence of use, use behaviours, and preferences, among 4,684 adults in Wave 1 of the survey in early 2018 [138], a time subsequent to the Japanese launches of IQOS in 2014 and both glo and Ploom TECH in 2016. The overall prevalence of current HTP use was 2.7%, and the prevalence of exclusive use was 0.9%. The majority of HTP use was found among current and former smokers, and non-smokers who were current users of HTPs formed a negligible (0.02%) proportion of the population. The majority of HTP users used them daily, and those who were cigarette/HTP dual users typically used significantly fewer HTP tobacco sticks each day. Age was a determinant of the likelihood of using a particular HTP, with IQOS being used by greater proportions of younger adults (aged 20-29) and being less popular among older age cohorts. Brand preferences did not appear to depend to any degree on sex, income, or education level. The same group used data from the 2018 ITC Japan wave to assess patterns of dual use of HTPs and cigarettes [139]. Dual users were found to be younger and wealthier than exclusive smokers. However, there were no differences in the frequency of smoking, the number of cigarettes smoked per day, and smoking cessation behaviours between the two groups. Compared to exclusive HTP users, dual users reported a higher frequency of non‑daily HTP use and, similar to the previous analysis by Sutanto et al. [138], used a lower number of HTP sticks per day. Almost all (94%) dual users smoked every day; 48% used HTPs and smoked daily; and 46% were daily smokers and non‑daily HTP users. These data perhaps underline the importance of assessing use behaviours, as well as the prevalence of use when seeking to determine the population health impact of HTPs.

Using a Japanese nationally representative sample of 2,121 male and 2,507 female Japanese survey participants in 2018, Kinjo et al. [140] found ever use of HTPs among 7.6% of the population and past 30‑day use in approximately 4% of the population. Both ever and past 30‑day HTP use were markedly higher among males than females, by an approximate factor of 4 in each case. Dual use of HTPs was found in approximately 4% of the population, and this was also concentrated among males. In male survey participants, ever use and past 30‑day use of HTPs were the highest among those aged between 20 and 29 and generally declined as age increased. The likelihood of having used HTPs increased as the length of time spent in education increased and was more common among those who were employed or students. These associations were similar for female participants, though of note the prevalence of HTP use among female students was zero [140]. In a similar population‑based Japanese survey conducted approximately one year earlier than the study by Kinjo et al. [140], Afolalu et al. [141] reported a prevalence of current use of IQOS of approximately 2%, and again use was concentrated among males, those of a younger age, those with a higher level of education, and those with a managing profession. IQOS use was not observed in the student population, though no definition of students was provided in the paper. In a further survey sample, in which participants were self‑reported current IQOS users, participants had almost exclusively used other tobacco and nicotine products before initiating IQOS use [141].

Two studies of note examined HTP use among specific subsets of the Japanese population. Myagmar-Ochir et al [142] assessed HTP use among 7,714 retail business workers in the service industry, finding an overall use prevalence of 3%. As reported in other studies, use was focussed among males and approximately twice as likely among office workers. The second study by Oya et al assessed HTP use among 1,317 practising dentists, the overwhelming majority of whom were male [143]. Among 112 current (past‑month) HTP users, all were male and the majority were aged between 40 and 59. Approximately two‑thirds of these users were IQOS users, and of note the predominant use was of a single brand of HTP. Also of note, the majority of exclusive HTP users were former smokers, and only a very small proportion (less than 1%) were never smokers. 

South Korea is another country where several manufacturers launched HTPs earlier than in many others, leading to a significant number of studies assessing HTP use within this country. Kim et al. [144] used various population‑based survey data sources, as well as tobacco sales data obtained from the Korean government, to integrate an overall assessment of HTP and cigarette use. Beginning in 2017, HTP sales rose from 78.7 million packs to 379.3 million packs in 2020. This was associated with a reduction in cigarette sales, with volumes falling from approximately 3.4 billion packs of cigarettes sold in 2017 to approximately 3.2 billion in 2020. Using the Korea National Health and Nutrition Examination Survey (KNHANES) data from 2018, one year after HTPs were introduced into Korea, current (past 30‑day use) was estimated among 4.4% of the population, and such use was approximately 10 times higher among males than females [145]. The prevalence of dual use was high, and dual use was associated with a lower likelihood of attempting to quit smoking [145]. This contrasts with the findings following an analysis of a further Korean cross‑sectional study, the Korea Community Health Survey [146], which showed no difference in intentions to quit smoking between exclusive cigarette smokers and cigarette/HTP dual users. This latter finding is perhaps explained by the findings from a focussed study reported by Kim et al. [147], in which participants agreed that there was no strong correlation between the use of HTPs and smoking cessation. The authors of this study concluded that HTPs have the potential to weaken both external and internal motivating factors for quitting smoking [147], which concurs with the findings of Kim et al. [145].

While cross‑sectional studies can provide important information concerning the prevalence of HTP use, as well as correlates of use and associations, they have limited use in determining sequences of events (e.g., the use of one tobacco product beginning before the use of another product) as exposure and outcome status are determined at the same time points. In this regard, longitudinal studies have greater utility and can determine actual transitioning behaviours and their correlates, as well as identify predictors of use. In one such Korean longitudinal survey study, Yi et al. [148] examined sociodemographic and other factors related to HTP use among the KNHANES survey participants who took part in follow‑up surveys in 2017 subsequent to their completing the main survey between 2015 and 2017. HTP use was associated with being middle‑aged, having a higher income and a higher education level, and having either past or current experience of using e‑cigarettes. These findings concur with other Korean studies, as does the finding that the level of motivation to quit smoking was not a predictor of future HTP use [148].

Regarding the specific use of IQOS in Korea, Kim et al. [149] assessed awareness, the prevalence of use, and experience in using IQOS three months after it was introduced into the Korean market. Among the convenience sample used, 114 were male and 114 were female. Awareness of IQOS was similar among either sex. However, both ever and current IQOS use were focussed on females, a finding which contrasts with the more prevalent use of HTPs among males found in other studies including some from Korea. IQOS use prevalence was very low among never smokers and never users of e‑cigarettes. It should be noted however that the small sample size may preclude any strong conclusions from being drawn due to the possibility of random errors being present in the study.

One final Korean study retrieved by the literature search assessed the use of HTPs in places in which they are prohibited (‘stealth use’) [150]. In an online survey from a nationally representative sample of 7,000 Korean adults, 574 (8.2%) were current HTP users. Stealth use was self‑reported by approximately 79% of HTP users, a level approximately 10% higher than the prevalence of stealth use of cigarettes but similar to stealth use of e‑cigarettes. Stealth use of HTPs was more common among daily HTP users, among those exhibiting a greater degree of dependence on HTPs as assessed by time to daily first use as an indicator of dependence, and among co‑users of HTPs, cigarettes, and e‑cigarettes. The reasons for the high prevalence of stealth use of HTPs could not be determined in the study.

HTP use has also been assessed in the Chinese population in Hong Kong, a Chinese administrative region in which HTPs are not yet marketed [151]. Perhaps unsurprisingly, HTP use prevalence was very low (1%), and what little HTP use existed was associated, similar to Korea, with higher income and educational attainment, but not with sex. Of note, former and current smokers had a 1.4 times and a 2.9 times greater likelihood, respectively, of reporting awareness of HTPs [151]. In another country, Mexico, a multi-wave study was conducted in 2018 and 2019 with a sample of smokers and e-cigarette users. This was a time when HTPs were banned and unable to be purchased. Approximately 17% of respondents were aware of HTPs and approximately 1.1% had used them [152]. Factors associated with interest in using HTPs were being an older adult (vs. being a younger adult, and particularly aged between 30 and 39), being a daily vs. a non‑daily smoker, and having recently made a quit attempt [152]. A more recent study was conducted by the same group between 2019 and 2021 when HTPs were available for purchase while a ban on HTP sales was undergoing judicial review [153]. Survey data analyses found an HTP use prevalence of 1.1% among Mexican smokers and e‑cigarette users [153], a finding which concurs both with the prior study [152], as well as the finding that HTP use remained constant between 2019 and 2021 [153]. Current HTP use in the more recent study was associated with being female, having a higher education level, with daily vs. non‑daily smoking, having recently made a quit attempt, and with the belief that HTPs are less harmful than cigarettes, although the latter association was not significant [153].

In Qatar, a country in which the prevalence of cigarette smoking is over 25% [154], HTP use was estimated as being found in less than 1% of the population using an adapted version of the Global Adult Tobacco Survey [154]. Of note, HTP use appeared more common among Qatari (vs. non‑Qatari) survey respondents. While this study also assessed factors associated with tobacco product use, these were not reported specifically for HTPs, likely due to the small number of HTP users in the survey sample.

Adult HTP Use in Europe

The marketing of HTPs is widespread across Europe, and a number of studies have examined the use of HTPs either in Europe in general or in individual European countries. Gallus et al. assessed HTP awareness and use in 11 European countries, representing 80% of the population, in 2017 and 2018 using face‑to‑face survey methodologies [155]. Among the approximately 11,000 respondents, almost three‑quarters were unaware of HTPs, approximately one‑quarter were aware but had not used them, 1.5% had tried them, and 0.1% were both former and current HTP users. Awareness and use differed between countries, with HTP use most common in Greece. Factors associated with a greater likelihood of HTP use across all countries combined were being male, younger aged, more highly educated, and being a former or current smoker/e‑cigarette user. Regarding cigarette smoking, former smokers were more than four times more likely, and current smokers were more than eight times more likely, to report ever use of HTPs [155]. In a more recent Europe‑wide study, Laverty et al. [156] assessed 2020 data from the Eurobarometer, a collection of cross‑country public opinion surveys conducted regularly on behalf of the European Union institutions since 1974. Findings were similar to those of Gallus et al. [155], although the slightly higher pan‑European ever use (6.5%) and current use (1.3%) reported by Laverty et al. [156] perhaps reflect the growth in HTP sales in the time between the two surveys. Similar to Gallus et al. [155], ever and current HTP use were associated with being younger, male, more highly educated, and being either a former or current smoker [156]. In a more focussed assessment of just six European countries between 2016 and 2018, Lotrean et al. [157] assessed data from the International Tobacco Control and Evaluation Project 6 European Country (ITC 6E) surveys. The findings were similar to those of Gallus et al. [155], with awareness and use found at low levels and the highest in Greece, and HTP use being associated with both daily smoking and past 12‑month attempts to quit smoking [157]. Specifically regarding Greece, Panagiotakis et al. [158] assessed HTP awareness and use more recently using survey data from a nationally representative sample of the Greek population in 2022. Exclusive HTP use was found in 4.6% of the population, and dual use of HTPs and cigarettes was found in a further 1.3%. Although no data were presented for HTPs alone (they were combined with e‑cigarettes in a single product category), use was associated with a higher level of education, being fully employed, and of younger age, and a past history of cigarette smoking. Interestingly, HTP use was also significantly associated with a decreased incidence of hypertension, as assessed from participants’ medical records [158].

Several studies have assessed HTP use in Poland. From a 2019 nationally representative survey, Pinkas et al. reported an overall HTP use prevalence of 1.9%, with a prevalence approximately equal among males and females [159]. Using data collected at approximately the same time, Jankowski et al. [160] reported a prevalence of ever HTP use of 8.5% among Polish physicians, which is higher than that found in the general population [159]. Daily HTP use among police employees has also been reported to be higher than that of the general population [161]. The most recent Polish data stem from an online, cross‑sectional, nationally representative survey study conducted in 2022 [162], in which the overall prevalence of daily HTP use was 4.0%, with use more common in males and those aged between 30 and 49 [162]. In another Eastern European country, Romania, Hussain et al. [163] reported a similarly low prevalence of current use of HTPs (1.3%), and HTP use was strongly associated with current and former, but not never, smoking.

In Germany, two studies have assessed HTP use. In the first study, Atzendorf et al. [164] used data from the 2018 Epidemiological Survey of Substance Abuse (Epidemiologischer Suchtsurvey, ESA), a nationally representative sample of 9,267 German adults. Past 30‑day use of HTPs was found in only 66 (1.3%) men and 15 (0.3%) women, giving an overall use prevalence of 0.8% [164]. In the second study, Kraus et al. [165] assessed data from 10 waves of the ESA survey study which collected data between 1995 and 2021. Past 30‑day use of HTPs was not found in any year among survey respondents aged between 18 and 24 and was only found in 2021 among a very small proportion (less than 1%) of respondents aged between 25 and 59 [165].

In Italy, a longitudinal survey study assessed the changes in tobacco and nicotine product use behaviour by collecting data from April to May and November to December 2020 [166], a period coinciding with the COVID‑19 pandemic. Among 2,122 baseline never smokers, 99 (4.7%) had started smoking at follow‑up and these individuals were more frequently e‑cigarette and HTP users at baseline. Compared with never HTP users, the relative risk (RR) of smoking initiation was 3.67 for former HTP users and 5.80 for current HTP users. While the RR for smoking initiation among never-smoking HTP users could be interpreted as a ‘gateway’ phenomenon, this transition from never smoking to smoking initiation via HTP use was only observed in approximately 1% of the total survey population. Also reported in this study, among 344 former smokers at baseline, 59 (17.2%) had relapsed at follow‑up. These individuals were more frequently HTP users (RRs were 2.51 for former HTP users and 3.32 for current HTP users). Among 719 current smokers at baseline, 614 (85.4%) were still smoking at follow‑up, and 105 (14.6%) had quit smoking. Participants continuing smoking were more frequently current HTP users (RR: 1.17), which concurs with the findings of other, cross‑sectional studies discussed earlier in this review of significant dual use of cigarettes and HTPs.

A number of studies have examined HTP use in England and the UK. In the first study, Simonavicius et al. surveyed smokers’ use of alternative nicotine products, including HTPs, NRT, and e‑cigarettes, in 2019 [167]. Among the 1,777 smokers surveyed, 1,281 (72.2%) had ever tried or used alternative nicotine products, but only 4.9% had ever tried a HTP, a value slightly higher than that found two years earlier in 2017 [168]. Alternative nicotine product trial or use was significantly associated with White ethnicity and lower socioeconomic status, which contrasts with the findings from other studies, daily smoking, higher motivation to stop smoking, higher numbers of cigarettes smoked per day, shorter time to first cigarette each day, and higher scores on the Heaviness of Smoking Index [167].

Another study assessed trends in HTP use among users of alternative nicotine products between 2016 and 2020 using data from the Smoking Toolkit Survey, a monthly cross‑sectional survey of a representative sample of adults in England [169]. This reported that HTP use was low (approximately 2%), though the prevalence of HTP use was slightly higher in 2016 and 2020. An intriguing finding from this study is that never smokers were twice as likely, and former smokers half as likely, to have used HTPs compared to current smokers [169]. One likely explanation for this is that the survey population comprises current users of alternative nicotine products and not the general population, which limits the generalisability of the findings. Cox et al. [170] assessed data collected from the general population in England (2020) from the Smoking Toolkit Survey. They reported extremely low values for the prevalence of ever, regular use of HTPs, which were 0.4% among current smokers, 0.2% among former smokers, and 0.1% among never smokers [170]. In a different, cross‑sectional survey study in the UK in 2019, the prevalence of HTP ever use was estimated at approximately 6% and HTP current use at approximately 3% [171]. Ever use was significantly associated with being younger, having some (as opposed to no) university education, living in Greater London, and using cigarettes/e‑cigarettes [171]. One study took an interesting and unique approach to assessing HTP use in the UK and examined changes in tobacco/nicotine product expenditure over time between 2018 and 2022 [172], also using data from the Smoking Toolkit Survey. Inflation‑adjusted expenditure on cigarettes grew by 10% from 2018 to 2020 and then fell by 10% from 2020 to 2022. These changes coincided with a 13% reduction in cigarette consumption and a 14% increase in the proportion of mainly smoking hand‑rolled cigarettes. Mean expenditure on alternative nicotine products, including HTPs, grew by 34.8% between 2018 and 2022, though, due to the low number of respondents who were exclusive HTP users, changes in expenditure on HTPs could not be specifically assessed [172]. Nevertheless, it is interesting to speculate on the possibility of an association between decreased cigarette consumption and increased consumption of alternative tobacco/nicotine products, potentially suggesting that the use of one causally displaces the other.

Adult HTP Use in the United States

In the US, HTPs are available for purchase and two products, IQOS and Logic Vapeleaf, have been commercially marketed in the US for a number of years albeit in small test markets [173] and the US Food and Drug Administration granted reduced exposure marketing approval for IQOS in July 2020. The earliest assessment of HTP use prevalence in the US arose from a study by Nyman et al. [174] in which data from the 2016 and 2017 Tobacco Products and Risk Perceptions survey studies were assessed. Between those two years, HTP awareness among adults increased from 9.3% to 12.4%, ever use rose from 1.4% to 2.2%, and current (past 30‑day) use increased from 0.5% to 1.1%. Among the ever and current HTP user populations, the proportion of survey respondents who were never smokers was very low (0.8% and 0.3%, respectively), and HTP users were significantly more likely to be either former or current smokers and either former or current e‑cigarette users. Current HTP use was also associated with short‑term smoking cessation intentions [174]. In a similar cross‑sectional analysis using data from the 2019 Tobacco Use Supplement to the Current Population Survey, Azagba et al. [175] reported a similarly low (0.51%) prevalence of HTP use among US adults, and again current use of cigarettes and e‑cigarettes were predictors of HTP use, as was living in a metropolitan (as opposed to a non‑metropolitan) area [175]. In a study using both cross‑sectional and longitudinal approaches to assess HTP use among young adults aged 21-24 in Hawaii, HTP use was associated with cigarette smoking and/or e‑cigarette use, with nicotine dependence, with motivations to quit smoking, and with the current use of e‑cigarettes for smoking cessation [176]. In a broader (six‑city) assessment of HTP use among US young adults in 2019 [177], HTP awareness and ever use were low (9.7% and 3.5%, respectively), and past‑year purchase of a HTP was found in only 2.4% of participants. Again, current cigarette smoking and/or e‑cigarette and other tobacco product use were significantly associated with HTP ever use, noting however that HTP use assessed in this study combined both electrically heated and carbon‑heated HTPs as a single product type [177].

In 2020, data were collected in the Health Information National Trends Survey (HINTS) 5 cycle 4, a nationally representative survey administered by the National Cancer Institute assessing US adults aged 18 and older [178]. HTP awareness was 14.8%, which is indicative of an increased awareness compared to earlier studies. Factors associated with a higher likelihood of awareness were being male, having a lower income, and being a smoker. The prevalence of ever use of HTPs was estimated at 2.2% of the population, and interestingly both awareness and ever use of HTPs were higher among individuals with moderate/extreme concerns about getting cancer [178], a finding which the authors propose may be used to hypothesise that HTP can be (mis)construed as a safer smoking alternative and a viable quitting method.

Data from the ITC Four Country Smoking and Vaping Survey have been used in some studies to assess HTP use in the US, as well as Canada, England, and Australia, among adult smokers and e‑cigarette users [179,180]. In the first study, Miller et al. [180] estimated HTP awareness in approximately 30% of the US smoker/e‑cigarette user population, a figure similar to the other countries assessed. Every trial of HTPs was observed in 2% of US adult smokers/e‑cigarette users, with values of 3.3%, 2.4%, and 0.9% estimated for Canada, England, and Australia, respectively. Current HTP use prevalence among smokers/e‑cigarette users was 0.7% in the US and 0.8%, 1.2%, and 0.2% in Canada, England, and Australia, respectively [180]. In the second study [179], current HTP use was found to be significantly more likely in England. Across all four countries, current HTP use was associated with higher socioeconomic status, with cigarette and e‑cigarette dual use, with the past 30‑day use of non‑combusted tobacco products, and past 30‑day use of cannabis [179]. Analysing data collected in 2020 from the ITC Four Country Smoking and Vaping Survey but focussing on US data among adults who smoke or use e‑cigarettes, Felicione et al. [181] estimated a 25.8% prevalence of HTP awareness, as well as 8.4% and 2.5% prevalence of HTP ever use and current use, respectively. Awareness and use were found to be higher among younger survey respondents and the highest in the 18-24 age group among those who currently or formerly smoke and/or use e‑cigarettes [181].

Analysing data from a Marijuana Use and Environment Survey administered to a representative sample of the US adult population between late 2019 and early 2020, Zhu et al. [173] reported an overall prevalence of HTP awareness of 8.1%. Both overall and regardless of sex, age, ethnicity, and education level, HTP awareness was higher among ever smokers and ever e‑cigarette users than among never smokers and never e‑cigarette users, respectively. In addition, ever and current use of HTPs was estimated in 0.55% and 0.1% of the population, respectively, and both ever and current HTP use were associated with a history of either smoking cigarettes or using e‑cigarettes [173]. In 2021, awareness, ever use, and current use, of HTPs were reportedly higher, at 23.6%, 8.9%, and 3.0%, respectively [182]. The difference in prevalence estimates reported previously using other data sources likely arises due to the study by Sparrock et al. [182] assessing US adults who are recent former and current commercial tobacco users and not the general population (e.g., Zhu et al. [173]). Current use of HTPs was significantly more likely among the White and Hispanic populations, among those using e‑cigarettes, hookah, and other combustible tobacco products and among those with a higher level of income [182].

In one final study of note, Levine et al. [183] assessed interest in, and use of, IQOS among adults in Israel and the US, noting that IQOS was launched in Israel in 2016 and in the US in 2019. Weighted current, former, and never IQOS use prevalence estimates among US adults were 1.1%, 1.9%, and 97.0%, respectively, and for the US population, current use of IQOS was more common in the Black and Asian sub‑populations, among those born in the US, and among those with a higher income level. IQOS use was also significantly associated with cigarettes, e-cigarettes, hookah, cigars, pipes, and smokeless tobacco use status. Similar associations with the use of other tobacco/nicotine products were also seen in Israel, and in that country being male was also a strong predictor of current use of IQOS, which differs from the US where sex was not a predictive variable [183].

Summary of Adult HTP Use Studies

In summary of this section, HTP use among adults was reported to be low. Similar to findings in youth and young adults, the majority of studies indicated that HTP use was extremely rare among never smokers. HTP use was generally associated with being younger and with having higher levels of income and education. In a similar trend as for youth and young adults, there were some clear differences between countries, regions, and cultures. In some studies, intention to quit smoking was found to be a predictor of HTP initiation, which aligns with the potential for HTPs to help smokers transition away from cigarettes and towards potentially less harmful forms of tobacco/nicotine use. One further potentially interesting point regarding adult use relates to the following section on the impact of HTPs on cigarette consumption. The study by Jackson et al. [172], supports the hypothesis that there is a population‑level association between reduced consumption of cigarettes and increased consumption of alternative tobacco and nicotine products, including HTPs. This hypothesis would need rigorous testing but, if correct, would suggest a strong role for HTPs in population‑level THR. It is also notable that adult HTP users, similar to the youth and young adult populations, reported a significant degree of dual and poly‑use of HTPs with one or more other tobacco and nicotine products including cigarettes. While dual use with a reduction in cigarette consumption would still elicit exposure reductions and therefore contribute to THR, and since a period of dual use could be part of a smoker’s journey towards complete abstinence, the nature of HTP and cigarette dual use (e.g., is it associated with reductions in cigarette consumption and/or future likelihood of complete abstinence, or is it prolonging smoking among those intending to quit) warrants further investigation. Of final note, several studies highlight that HTP use does not provide a ‘gateway’ effect into cigarette smoking among never smokers, a use pattern which would be detrimental to THR.

Impacts on Cigarette Smoking Including Smoking Reductions, Complete Transitioning, and Relapse

Emissions from HTPs contain significantly fewer, and substantially lower levels, of harmful chemicals compared with cigarette smoke [10-14]. This translates into reduced toxicant exposure in smokers who switch to using HTPs [31-37]. Some studies have suggested that this further translates into beneficial changes in some biomarkers of potential harm, even with relatively short‑term transitioning [31,38-41]. Since HTP use may potentially therefore pose a lower disease risk than cigarette smoking [19], the availability of HTPs could offer population‑level benefits if they serve as an acceptable alternative form of nicotine delivery for current smokers and support their complete transition away from cigarette smoking. This section reviews articles related to transitioning and relapse, with a view to examining the population‑level impact of HTPs.

Asian Studies on Smoking Impacts

Since South Korea is a country in which HTPs were launched before many other countries, a number of studies have assessed the impact of HTPs on smoking behaviour in Korea. The earliest paper published in Korea was in 2019 and arose from a survey study assessing HTP use and smoking patterns among 21,100 Koreans in 2018 [184]. In the survey population, approximately 2% were current (past 30‑day) HTP users, noting that at the time only IQOS was available in Korea. Among the HTP users, the prevalence of the ever use of HTPs among never smokers was 0.05%. Over 96% of HTP users were dual-using HTPs with cigarettes, taking into account the triple use of HTPs, cigarettes, and e‑cigarettes, while only a very small proportion (less than 2%) were exclusive HTP users. Overall, this led to the suggestion that HTPs were complementing, and not a substitute for, cigarette smoking. Additionally, stability in total tobacco sales (cigarettes and HTPs combined) between 2014 and 2020 in South Korea is suggestive more of dual use than of complete transitioning [185].

Based on analyses of the 2019 KNHANES data, Yu et al. [186] assessed tobacco use and cessation behaviours among adult single and dual users of cigarettes and HTPs. In terms of smoking cessation, no statistically significant differences between exclusive smoker/exclusive HTP user/dual user groups were seen for attempting to quit tobacco use. Of concern perhaps, exclusive HTP users and dual users were less likely to be in the ‘preparation’ stage of quitting tobacco use, although this relates to quitting the use of HTPs, as well as stopping smoking.

Yun et al. [187] assessed smoking cessation among current triple users of cigarettes, e‑cigarettes, and HTPs, among 5,239 mostly male Korean adults. Data from this cross‑sectional survey suggest that, among these triple users, the most common behaviour change was to continue such use, followed by stopping e‑cigarette and HTP use to become an exclusive smoker. Only a small proportion of the triple users (approximately 7%) quit smoking, either alone, by also quitting e‑cigarette use, or by stopping nicotine/tobacco use altogether. In a similar study, but this time examining dual users of cigarettes and HTPs, Won et al. [188] also examined quitting behaviours among Korean adults. Exclusive HTP users were significantly less likely to have future cessation plans and to have made cessation attempts, noting though that this relates to quitting HTP use rather than quitting smoking. In this regard, in both studies [187,188], given the way in which the analyses were performed, it is difficult to determine the impact of HTPs on smoking cessation.

Lee et al. [189] assessed associations between the frequency and quantity of HTP use among a representative sample of 2,470 Korean adolescents. Approximately 20% of the study cohort were HTP users and participants’ frequency and quantity of HTP use were generally associated with their frequency and quantity of cigarette smoking. However, the frequency and quantity of HTP use were not associated with attempts to quit smoking. Additionally, similar to other studies described previously, compared to exclusive smokers, triple users were less likely to attempt to quit smoking [189]. A further Korean study conducted in 2019, a time when HTP sales accounted for over 10% of all tobacco sales, examined the association between the use of new tobacco and related products and cessation behaviours [190]. Among 2,831 participants in the Tobacco and Health in Korea (THINK) survey, 26% were exclusive smokers, 13% were exclusive HTP users, 14% were dual‑using cigarettes and HTPs, and 12% were triple users (cigarettes, HTPs, and e‑cigarettes). After adjusting for confounders, triple users were 1.4 times more likely to have attempted to quit smoking in the past year compared with exclusive smokers, while dual (HTP and cigarette) users were no more likely. In addition, exclusive HTP users were less likely to be both ready to quit HTP use and to have made quit attempts compared with exclusive smokers, and this is likely related to a further finding that exclusive HTP users were more likely than smokers to perceive HTP as being less harmful than cigarettes [190]. The relationship between quitting smoking and HTP use was further assessed in Korean adults by Kim et al. [191]. Among 1,530 current tobacco users, approximately half were current smokers, 5% were current HTP users, 19% were cigarette and HTP users, and 12% were triple users, which is again indicative of a large degree of HTP co‑use rather than exclusive use. The likelihood of past‑year quit attempts was similar among smokers and exclusive HTP users but was significantly higher among HTP and e‑cigarette dual users.

The most recent South Korean study assessed attempts and plans to quit tobacco product use among 1,288 exclusive, dual or triple users of HTPs, cigarettes, or e‑cigarettes in 2022 [192] and made determinations of predictors of quitting plans and attempts, including domains such as health concerns and conditions, stress, and addiction. Among single users, health behaviours such as eating healthily or having regular health checks, and time to first tobacco use in the morning, were positively related to past‑year quit attempts, and addiction was negatively related to quit attempts. In dual users, quit attempts were significantly related to health behaviours but were negatively related to stress or addiction. Among triple users, none of the factors predicted quit attempts. Regarding plans to quit using tobacco products, among single and dual users quitting plans were positively related to the time to first tobacco use in the morning while in single users, the self‑rated addiction scale was negatively related to quitting plans. These are useful observations, but in terms of assessing HTP quitting plans and attempts, no data were presented for HTP users in isolation.

Other studies have assessed the role of HTP use in smoking cessation in Japan, as well as a market in which HTPs were launched prior to their more widespread availability. Kanai et al. [193] prospectively assessed the association between HTP use in Japanese workplaces and cessation rates. Cessation was defined as stopping the use of all nicotine‑containing products, noting that in Japan this means tobacco products since products containing nicotine alone are classed as pharmaceuticals. Among 158 current tobacco users, 28% achieved tobacco cessation after following an intervention protocol. Baseline HTP use was not associated with any greater success in quitting compared with no use at baseline, while pharmacological support (NRT or varenicline) did increase cessation outcomes. A prospective design was also used by Harada et al. [194] in their assessment of changes in smoking habits in Japan while estimating health impacts by assessing a marker of lung function (FEV1, the forced expiration volume over one second) among study participants. The prevalence of exclusive HTP and HTP dual users was 0.8% and 0.6%, respectively, in the resident population and 5.0% and 1.9% in the worksite population, respectively. Smoking prevalence was 8.9% in the residents and 8.6% in the worksite population. Among exclusive smokers and exclusive HTP users, the number of cigarettes smoked per day at baseline and the number of tobacco products used per day at follow‑up followed a decreasing trend, while in dual users it increased due to greater HTP use despite lower cigarette consumption. In the resident population, logistic regression analyses of the difference in annual FEV1 change compared to an exclusive smokers control group showed that exclusive HTP users had a moderately slower decline in FEV1, dual users had a worsening decline, and never and former smokers had a much slower decline.

Adamson et al. [195] conducted a survey study focussing on three cities in Japan (Tokyo, Osaka, and Sendai), which were either the largest metropolitan areas in Japan or test markets for HTP launches. Among the 4,154 participants, 779 were current HTP users, and the reasons stated for such use were mostly to reduce harm to themselves and people around them. Exclusive use of HTPs was more common among younger participants, and complete transitioning was observed in 5% of participants who were exclusive cigarette smokers 12 months prior to the study. Only 10 HTP users were never smokers. Complete transitioning was slightly more common in women than men and the highest in the youngest age group (25-29 year olds) [195]. In a further early study in Japan, sales data were used to assess the impact of the introduction of IQOS on cigarette sales [196], which appeared to show reductions in cigarette sales associated with IQOS introduction that was similar in degree, but slightly time‑shifted, in regions with differences in when IQOS was launched. While these data do not provide any information on the exclusive or dual use of IQOS with cigarettes, they are an indication at least that IQOS could, at the time and in Japan, displace cigarette sales. This possibility is perhaps disputed however based on longitudinal survey data from 7,044 Japanese adults in which smoking cessation and relapse at a one‑year follow‑up assessment were determined in relation to current HTP use at baseline [197]. At baseline, 17%, 9%, and 6% of the survey participants were current smokers, HTP users, and dual users, respectively. Among current established smokers, HTP use was significantly associated with a decreased likelihood of ≥ 1-month cessation, while among former smokers, HTP use was associated with smoking relapse within those who last smoked more than one year ago. Both these findings led the authors to conclude that HTP did not help smokers quit or prevent former smokers from relapsing.

Nomura et al. [198] used a retrospective cohort study design to assess the outcomes of a telemedicine smoking cessation programme in Japan, comparing smoking cessation rates between exclusive smokers and either exclusive or dual HTP users between 2018 and 2020. The proportions of participants within each user group were 52% exclusive smokers, 31% exclusive HTP users, and 16% dual users. The exclusive HTP users were typically younger, had smoked for fewer years, and had made more attempts to quit smoking. Continuous abstinence rates at week 24 were higher among exclusive HTP users (67%) than exclusive smokers (54%). This impact was also observed at week 52, although the difference was smaller in magnitude. In contrast, dual users had lower abstinence rates both at week 24 and at week 52. As with other studies, this study assessed complete nicotine abstinence rather than smoking abstinence specifically. Matsuyama et al. specifically assessed cigarette smoking relapse and initiation among adult former and never-cigarette smokers in the JASTIS Japanese prospective cohort study [199]. At baseline in 2019, 5% of participants self-reported using HTPs either almost every day or sometimes. Of these baseline HTP users, only a small proportion (12%) were never smokers, and larger proportions were smokers who had quit smoking recently (less than one year ago; 32%) or had quit smoking less recently (more than one year ago; 55%). HTP use was more likely among participants who were middle-aged, men, with high income, married or divorced/widowed, having a household member who smokes tobacco, and alcohol drinkers. At a one‑year follow‑up in 2020, HTP users were more likely to have relapsed to/initiated cigarette smoking, and 13% of HTP users at baseline had become cigarette smokers one year later. Only six participants who were never‑smoking HTP users at baseline had initiated smoking at the follow‑up. When this number was placed into the context of the whole survey population, the initiation risk of HTPs was extremely low (0.15%). In former smokers, HTP use was not associated with relapse among recent former smokers but was associated with relapse among longer‑term former smokers. However, when this is also placed in the context of the whole survey population of former smokers, only 33 of the 2,036 former smokers (1.6%) had relapsed due to HTP use.

Two studies examined the association between the use of HTPs and smoking cessation outcomes, in both youth [200] and adults [201]. In the prospective youth cohort study by Xia et al. [200], 579 smokers aged ≤25 years in Hong Kong who were willing to receive telephone counselling for smoking cessation were enrolled and followed for a period of six months to assess cigarette abstinence and smoking reductions. Self‑reported seven‑day abstinence was almost twice as likely among non‑users of HTPs than among HTP users at six months. Furthermore, HTP users were less likely to be prepared to quit within the next 30 days. No significant differences in cigarette consumption were observed between the two groups. In the adult prospective cohort study among 1,213 verified smokers in Hong Kong [201], current HTP use at baseline was not associated with cigarette abstinence at both three and six months. Overall data from these two Chinese studies are consistent and do not show an appreciable transitioning potential of HTPs, and among youth, these data suggest that HTPs may prolong cigarette smoking and reduce abstinence rates.

European Studies on Smoking Impacts

Outside Asia, other early HTP adoption data have come from Italy, a European market in which HTPs were launched prior to many other markets. Gallus et al. [202] reported that, among 3,000 adults representative of the general population, 49 were ever HTP users and of these, 9.1% started or re‑started smoking cigarettes, and 14.6% quit smoking after initiating HTP use. The paper also reports some trends in smoking/tobacco use behaviour such that declines in smoking prevalence were halted in 2018/2019 and that overall tobacco consumption rose in this period. Whether these were due to the introduction of HTPs into Italy, or some other extrinsic factor, is not clear. Carreras et al. [203] reported a sharp rise in HTP use between 2018 and 2021, using data from the cross‑sectional annual Progressi delle Aziende Sanitarie per la Salute in Italia (Progress of the Health Trusts in Italy, PASSI) survey conducted in representative samples of adults aged 18-69. In this period, HTP use grew from 0.5% in 2018 to 2.5% in 2021, and approximately three‑quarters of HTP users were dual users with cigarettes. The use of HTPs by never smokers also rose during this time, though at the end of the sampling period such use was seen in only less than 0.5% of the population. From a statistical perspective and compared to cigarette smokers, HTP use was significantly less likely among former smokers (ORs: 0.43-0.75, depending on the age group), and highly unlikely among never smokers (ORs: 0.03-0.06) [203]. In another Italian study, Caponnetto et al. [204] reported data from a two‑week randomised noninferiority switching trial to compare effectiveness, tolerability, and product satisfaction between IQOS 2.4 Plus and a refillable e‑cigarette among 211 smokers not intending to quit smoking. A quit rate of approximately 40% was seen for IQOS and, at week 12, a seven‑day point prevalence of smoking abstinence of over 50% was reported. In addition, among those who did not quit smoking completely, reductions in cigarette consumption were observed, with an average reduction of almost 50%. The quit rate in this controlled study [204] was not dissimilar from that observed in the general population [203], and while dual use was also observed, this came with a significant decrease in the number of cigarettes smoked.

In a Swiss study, Queloz et al. [205] assessed dependence and cigarette withdrawal symptoms, two factors that may be associated with the successful uptake of, and substitution of cigarettes with, reduced harmful products, such as HTPs [47,206,207]. Among 139 IQOS users, all of whom were current or former smokers at the time of initiation of IQOS use, half reported that they were less dependent (assessed using a modified version of the Fagerström Test for Cigarette Dependence [208,209]) on IQOS than cigarettes, 44% were equally dependent on both products, and 5% were more dependent. Regarding cigarette cravings among respondents who experienced them, 84% found that IQOS relieved it “a lot” to “totally”. Both these findings are indicative of a potential role for IQOS in acting as a smoking substitute. In a study conducted in four Arabic countries (Saudi Arabia, Egypt, Kuwait, and Yemen) in 2021 and 2022, Alanazi et al. [210] assessed cigarette smoking behaviour (quitting attempts and desire to quit cigarette smoking, nicotine dependence, and consideration of transitioning to nicotine products with reduced health risks), and awareness of, use of, and susceptibility to use of, HTPs. Awareness of HTPs was low, averaging 24% across the four countries, and the likelihood of ever making a quit attempt was significantly higher among lifetime and current HTP users, with ORs of 2.6 and 1.8, respectively. These results are suggestive of the role of HTPs in aiding smokers with an interest in quitting to transition away from cigarettes. 

Studies on Smoking Impacts in the US

Three studies examined smoking behaviours among youth and adults in the US in order to understand potential changes in behaviour associated with HTP use. Brown et al. [211] assessed the associations between the prevalence of smoking quit attempts in US adolescents and awareness and use of e‑cigarettes and HTPs using data from the 2019 NYTS survey. Approximately 17% of participants were aware of HTPs, and ever HTP users made up only a small proportion (less than 3%) of the 19,018 survey respondents. Regarding the 398 HTP ever users, they were no more likely to have made tobacco quit attempts than HTP never users, either when HTPs were used exclusively or when dual-used with e‑cigarettes. Similar findings were reported for those aware of HTPs. Another study assessed the use of non‑cigarette tobacco and nicotine products in four countries, including the US as well as Australia, Canada, and England, by analysing the data from the 2020 ITC Four Country Smoking and Vaping Survey [212]. Across all four countries, the average prevalence of current HTP use was 3.5%, and within the individual countries, this prevalence ranged from 1.1% in Australia to 4.2% in both England and Canada. In the US, the current HTP use prevalence was 2.7%. HTP use across all four countries (these data were not broken down by country) was significantly less common, by a factor of 2, among recent ex‑smokers compared with current smokers. This suggests a higher prevalence of dual use than exclusive use of HTPs, and the authors comment that any transition away from smoking to using alternative nicotine products, including HTPs, can take time with a period of experimental use common before any complete switch, or abandonment of it as a viable alternative [212].

In a prospective pilot study, DeAtley et al. [213] examined the role of IQOS risk perceptions in smoking behaviours among 27 daily, cigarette smokers in Philadelphia, USA, who were interested in quitting within the next six months. Participants were introduced to IQOS and then completed assessments of risk perceptions. Participants were given an IQOS device, charger, and IQOS consumables (HeatSticks) and asked to switch completely from smoking to using IQOS for 14 days. The effects of risk perceptions on changes in IQOS use, cigarettes per day (CPD), the substitution of IQOS for cigarettes, and motivation to quit smoking were evaluated. Over the 14‑day switch period, CPD decreased significantly from an average of approximately three CPD to an average of 0.33 CPD at day 14. IQOS use increased significantly, from an average of eight to an average of 10 HeatSticks per day, and the percentage of IQOS HeatSticks that replaced CPD also increased significantly. Perhaps counterintuitively, participants who perceived IQOS as less risky than cigarettes used fewer HeatSticks per day. However, a lower percentage of IQOS HeatStick substitution for cigarettes was observed for participants with higher vs. lower risk perceptions. Motivation to quit smoking also increased from baseline to day 14, although differences in risk perceptions were not predictive of changes in motivation. Overall, this study perhaps suggests a role of better-informing smokers about the RR of HTP use compared to cigarette smoking in order to promote higher rates of transitioning. After all, we understand from studies assessing other reduced-risk smoking alternatives that negative perceptions are associated with reduced uptake and reduced likelihood of complete transitioning [52,58-61].

Summary of Studies Assessing the Impacts of Cigarette Smoking 

In summary, regarding behaviour change among smokers, many studies have demonstrated, at least in some individuals, the ability of HTPs to act as substitutes for cigarettes and to assist in complete transitioning among those who were current smokers at the time of HTP initiation. That said, a large amount of literature suggests a high level of dual use of HTPs with cigarettes. This concurs somewhat with findings from medium‑term clinical switching studies which have demonstrated complete transitioning rates of approximately 65‑70% [31,40], as well as some literature reviewed here suggesting lower intentions to quit smoking among cigarette and HTP dual users. Furthermore, a recent Cochrane systematic review [214] concluded that it remains uncertain whether HTPs help people stop smoking cigarettes based on an insufficient number of studies to date, a statement which aligns with the various findings of dual use in studies assessed in this review. While dual use may elicit reductions in exposure to the HPHCs and other toxicants found in cigarette smoke, depending of course on the degree of dual use and of any reductions in cigarette consumption, such use does not convey an optimal level of individual‑ and population‑level THR. The reasons for HTPs supporting dual use but not complete transitioning among some individuals are unclear, and certainly this area merits further studies to assess the reasons with a view to improving complete transitioning rates. Additionally, some (but not all) studies have uncovered a greater propensity to stop all tobacco/nicotine use among triple users of cigarettes, e‑cigarettes, and HTPs. Again, the reasons for this observation relating to triple use are unclear and could be a focus of future research efforts. Overall, the drivers of dual use, and whether dual use prevalence changes over time as newer products are developed or as awareness and perceptions of HTP changes, should continue to be monitored, given its importance in determining the population‑level THR potential of HTPs.

Drivers of Use, and Reasons for Use, of HTPs

As part of an overall assessment of the population‑level health impact of HTPs, it is important to consider reasons for use among HTP users. The earliest assessment in this area came from a study by Queloz et al., who used an online survey methodology to assess reasons for use, modes of use, perceived advantages, and perceived risks, among 102 Swiss users of an unspecified brand of HTP [215]. Approximately half (57%) of these users were current cigarette smokers, the rest were former cigarette smokers. Among the current smokers, four‑fifths were currently trying to reduce their cigarette consumption and approximately one‑third were trying to quit smoking. The HTP was reportedly used mainly to replace cigarettes (94%), because it was perceived to be less toxic than smoking tobacco (89%), to help stop smoking or to avoid starting smoking again (72%), to reduce cigarette consumption (71%), and because of not wanting to smell of cigarette smoke (71%). Regarding cigarette consumption, current smokers who were daily or occasional HTP users reported smoking a median of eight CPD, compared with 20 per day before initiating HTP use. This concurs with previous findings described above of HTPs aiding smoking reduction in the absence of complete transitioning. Among the former smokers, approximately two‑thirds reported that HTP use had helped them stop smoking, which also concurs with studies described previously suggesting the utility of HTPs in facilitating a complete transition away from cigarette smoking. The reasons for regular use of HTPs among current and former smokers were also assessed using data from the 2018 Japanese ITC survey [216]. Among 658 current HTP users surveyed, 549 were cigarette dual users and 109 were former smokers, suggesting a higher level of dual use in Japan than that seen by Queloz et al. [215]. Similar to that study though was the prime reason for use among current smokers, a belief of reducing harms, with 88% and 84% believing that HTPs were less harmful than cigarette smoking to themselves and others, respectively [216]. Other main reasons for use in current smokers were personal enjoyment (75.2%), more acceptable to others (72.5%), and that family or friends use them (58.9%). These reasons for use were also similar among former smokers. Compared to current smokers, former smokers were more likely to cite the reason that HTPs are less harmful to themselves or people around them, stress reduction, and taste. In contrast, current smokers were more likely to report that they could use HTPs in places where smoking is banned and to make socialising easier.

In a similar study from the same group, Korean ITC survey data were used to assess reasons for HTP initiation and use among 1,815 current and former smokers [217]. Comparable to Japanese HTP users [216], the majority of Korean HTP users (1,650) were dual users with cigarettes, while 165 were exclusive HTP users [217]. The most common reasons for initiating HTP use among all HTP consumers were out of curiosity (59%), family and friends use HTPs (46%), and they like the technology (35%). The most common reasons for regularly using HTPs among all HTP consumers were that they smell less than cigarettes (71%), they are less harmful to their own health than smoking (49%), and the stress reduction they bring (47%). Overall, 36% of dual users reported using HTPs to help quit smoking, 15% to reduce smoking but not to quit, and 49% for other reasons besides quitting or reducing smoking. Interestingly, some significant differences were observed between dual and exclusive users for some reasons for use. For example, exclusive HTP users were significantly more likely to report the personal benefit of being “less smelly” than cigarettes compared to dual users.

In a qualitative interview study conducted in the UK in late 2018 and early 2019, Tompkins et al. [218] assessed reasons for initiating use and reasons for continuing or stopping use, among 22 current and eight former IQOS users. Six main factors appeared to influence initiation and use of IQOS, which were related to health (wanting to reduce/quit smoking and perceptions of reduced harm), financial reasons (high start‑up costs but cheaper ongoing costs than smoking), physical reasons such as satisfaction and sensorial experience, practical reasons such as the ability to use in smoke‑free places, psychological drivers including similarities in rituals and routines between cigarette smoking and IQOS use, and social reasons such as improving social interactions. Another UK qualitative study assessed drivers and barriers to the use of novel nicotine delivery devices after the first use of either an HTP or an e‑cigarette [219]. Five main themes were identified: health knowledge, availability of and accessibility to novel products, cost, social acceptance, and use experience. There was curiosity and interest in the uptake and use of novel products, but the absence of reliable health effect information was identified as a barrier. Throat discomfort and high anticipated cost were among additional barriers identified for both the HTP and the e‑cigarette. In a similar, Lebanese, qualitative study of young adults aged between 18 and 30 who were familiar with e‑cigarette products [220], similar themes regarding determinants of HTP use emerged, including lower health risks, social enhancement and acceptability and a sense of community, positive sensorial experiences, positive affects including relieving stress, and availability, convenience and ease of use. Potential negative health impacts of HTP use were also identified as a reason not to use HTPs, as was addictiveness.

Two studies examined reasons for the use of HTPs among patients diagnosed with diseases associated with cigarette smoking and who, therefore, may benefit from transitioning to reduced-risk tobacco products as a means of minimising further harms or reducing symptom exacerbations. In one study, Isaji et al. assessed the actual use, and reasons for the use, of HTPs in 195 smokers with rheumatoid arthritis [221]. Almost half (44%) of HTP users with rheumatoid arthritis had considered using HTPs due to their disease, and 42% of them felt that using HTPs relieved disease symptoms. Among smokers with rheumatoid arthritis, the main reasons for HTP use were less smoke and odour (53%) and less harmful to health (24%). These proportions were similar to those reported by smokers without rheumatoid arthritis. A greater proportion of HTP users than cigarette smokers with rheumatoid arthritis stated that cigarettes are more harmful than HTPs, and approximately one‑third of HTP users reported that their cigarette consumption had decreased, or will decrease, upon HTP initiation. Additionally, the study reported that smokers with rheumatoid arthritis were more than twice as likely to use HTPs than smokers without the disease. Furthermore, the presence of comorbidities, including atopic dermatitis, psoriasis, dyslipidemia, hyperuricemia/gout, cardiovascular disease, hypertension, diabetes, liver disease, kidney disease, COPD, and cancer, were significantly associated with HTP use. In the second study among 411 COPD patients [222], those using HTPs were significantly more likely to be younger (average age approximately 65 among those who transitioned to HTP use compared to 74 among those who did not) and tended to be educated to a higher level. No significant differences were found for other clinical factors such as smoking pack‑years (a marker combining the duration and degree of cigarette smoking), appetite score, anxiety/depression, and lung function.

In one final study, Roulet et al. [223] assessed the predictors of HTP use in an actual use study among current, daily smokers recruited in a number of US states. Stemming from this real‑world assessment, primary predictors of HTP adoption were positive sensory assessment and ease of use, while socio‑demographic characteristics (e.g., age, ethnicity, sex) and smoking habits appeared much less important, although participants who smoked on average less than 21 CPD had a lower likelihood of HTP adoption than those smoking between one and 10 CPD [223].

Summary of Drivers of Use, and Reasons for Use, of HTPs

Overall, reducing the health impact of cigarette smoking, both to themselves and to others around them, appears to be a major driver of initiating HTP use, as does wanting to reduce cigarette smoking or quit completely. In contrast, having concerns about potential health risks of HTP use was seen as a barrier to initiation. Reducing other non‑health impacts, such as reducing the smell associated with smoking cigarettes, may also be a driver for use. Although the number of studies assessed in this section is small, dual use of HTPs with cigarettes was apparent in some studies, mirroring that discussed in earlier sections of this review. One other aspect of reasons for use that comes out of some papers reviewed is information concerning the RR profile of HTPs compared to cigarettes, with misperceptions being a barrier to uptake. This barrier needs to be broken down in order to encourage more smokers to consider switching to potentially reduced-harm tobacco products such as HTPs. This could involve educating smokers, policymakers, and regulators about the reduced harm potential of non-combustible nicotine delivery products. This has been proposed for other potentially reduced-risk products [224] and would potentially enable smokers to consider both the absolute and RR of HTP use and cigarette smoking and assist them in making an informed choice. Sociodemographic differences in HTP uptake may also need to be addressed for the same reason. Interestingly, reasons cited for initiating the use of HTPs exhibit some degree of regional differences, which may reflect cultural distinctions but also may reflect variations in harm reduction messaging concerning HTPs in different countries.

Population Modelling

Population modelling is a means by which data on the individual-level health impact and population‑level use behaviour can be integrated to form an overall population health risk assessment. Depending on the type of model being used, these models can predict future impacts on mortality, either in general or related to specific diseases, and their use typically relies on inputs/assumptions from non‑clinical and clinical studies, as well as tobacco use transitions data for both the candidate product and other tobacco products, such as cigarettes [225,226]. Max et al. [227] evaluated the Population Health Impact Model (PHIM) developed by PMI and used as part of an overall population risk assessment for IQOS filed with the US FDA under the MRTP programme. This study considered two key components of the model: the assumptions implicit in the model (which outcomes were included, relative harm of the new product compared to cigarettes, tobacco‑related diseases considered, whether dual or poly‑use of IQOS was adequately modelled, and what other tobacco products were included), and the data used to estimate and validate model parameters (transition rates between non‑smoking, cigarette‑only smoking, dual use of cigarettes and IQOS, and exclusive IQOS use; and starting tobacco use prevalence). For context, PHIM is a dynamic state transition model which models the impact of cigarette smoking and IQOS use on mortality due to four smoking‑related diseases by following a hypothetical cohort aged 15 and older for 20 years, and in which each individual is separately followed over time using pseudo‑random numbers to determine transitions between product states [228]. Inputs to the PHIM consist of prevalence (‘P’) and epidemiological risk (‘E’) components [229]. While the findings from this exercise suggest that the model underestimates mortality, partly due to transition rate assumptions, no actual mortality estimates were provided by the authors [227], and as such, it is challenging to determine how the population health impact of IQOS differs from that calculated by PMI. The PHIM has also been used to examine the population health impact of the introduction of IQOS into Japan, using in‑country estimates of uptake and transitioning, as well as clinical data [230]. The overall reduction in tobacco‑attributable deaths from lung cancer, ischaemic heart disease, stroke, and COPD for men and women combined was estimated to be 269,916 over a 20‑year period if tobacco use disappeared completely at baseline. In contrast, reductions ranging from 167,041 to 232,519 deaths were estimated if IQOS totally replaced smoking at baseline, based on the assumption that the sole use of IQOS approximated 70%-90% of the effect of quitting (i.e., the residual risk among those transitioning to IQOS was between 10% and 30%). When IQOS uptake rates consistent with market data were inputted into the model, the reductions were still substantial and ranged from 65,126 to 86,885 deaths, figures that were not altered significantly during sensitivity analyses.

Using a different type of model, a systems dynamics model, the health impact of the introduction of an HTP and an e‑cigarette into the Italian market was examined [231]. This analysis assumed that the Italian population behaved similarly to the Japanese population regarding HTP use since Japanese transition data were used as inputs into the model [195], though it is unclear whether such an assumption is valid given the differences between the two countries. The dynamics model initially represented traditional smoking populations (never, current and former smokers), which were calibrated using historical data, and these were then extended to include scenarios in which both cigarettes and other products (HTPs and e‑cigarettes) coexisted, giving rise to subpopulations of current and former users of both cigarettes and those novel products [232]. Changes in use statuses, such as product initiation, transition to exclusive or dual use, and cessation, were represented as flows between product use statuses, such that any individual could fall into one or more use categories (initiated, current user, quit, or relapsed) for any of the products. For each use category, specific mortality risks were estimated. Based on Japan's transition data and using an annualised reduction in smoking initiation of 3%, the HTP scenario projected a fall in smoking prevalence from 19.7% in 2010 to 0.8% in 2100, which compares with 2.9% if the HTP was not introduced into the market. The model further estimated that this would lead to a reduction of 9.9 million life years lost over the modelled 90‑year period, the majority of which were estimated to be saved in later years.

Overall, the number of population modelling studies conducted to estimate the potential impacts of HTP use is limited. However, the use of different models with different inputs and assumptions lead to each study identifying a clear population‑level health impact of HTPs, both in terms of reduced smoking prevalence and reduced mortality. Given the low number of studies, further work is necessary to utilise these powerful modelling tools to assess population impacts, particularly when considering the constant changes in perceptions, uptake, use, and impacts on smoking rates, of HTPs described earlier in this review.

Summary and suggestions for future research

The potentially reduced risk profile of HTPs relative to cigarettes is becoming increasingly clear, with HTPs presenting substantially lower exposure to HPHCs and other toxicants compared to cigarette smoke [31-37]. This profile potentially leads to favourable changes in various health indices among smokers who completely switch to HTP use [31,38-41]. What is less clear, however, is whether this differential risk profile at the individual level supports THR at the population level. Two main factors can be used to assess the population‑level risk of novel tobacco products such as HTPs. The first factor is the ability of HTPs to appeal to and be used by current smokers, particularly those who are otherwise uninterested or unwilling to quit smoking, since this may facilitate smokers’ complete transition away from cigarette smoking [47]. The second factor is the appeal of HTPs and their use among unintended populations, such as never smokers, as a means of initiating tobacco product use, particularly among youth who are potentially most susceptible to tobacco product use initiation [48]. The overall aim of this review was to assess both of these factors using data from behavioural studies to determine whether the individual-level THR potential of HTPs is being manifested at the population level. The key findings from our scoping review of the literature in areas of importance to THR are presented in Table 1.

**Table 1:** Key findings of the scoping review. Abbreviations: HTP, heated tobacco product; THR, tobacco harm reduction

Area	Finding
Individual-level risk	HTPs are potentially reduced-risk products for smokers who completely switch to using them [19,41-46]
Intended use	HTP use primarily confined to adult current and former smokers
Unintended use	Prevalence of use among never smokers, and among youth/adolescents/young adults, is very low; very little evidence of any ‘gateway’ effect
Switching potential	Association between reduced cigarette consumption and increased HTP consumption; a significant degree of dual-use needs further investigation, including whether it is a transitioning phase to complete switching
Access and awareness	HTPs available in an increasing number of countries, and awareness is also increasing
Perceptions	Widespread misperception of HTP use risk relative to smoking, which may undermine THR potential; need for better communication of risk targeted towards adult smokers, as well as policymakers, regulators, and the healthcare community

Based on the reviewed literature, HTPs are most commonly used by those with a history of smoking cigarettes, and the use by never smokers was very to extremely low, depending on the country. This low level of use was broadly seen across the youth, young adult, and older adult populations, although there is some evidence that HTP use may be increasing in each of these populations. Various drivers of HTP use were uncovered in a number of studies, and these included wanting to reduce the risks to health of smoking cigarettes both to themselves and those around them or wanting to stop smoking completely. One area that warrants further study is the observation of a significant proportion of dual use (i.e., cigarettes and HTPs). The largest benefit from transitioning to using HTPs likely arises due to complete transitioning away from smoking as this would elicit the largest possible reductions in toxicant exposure and changes in health effect indices. It is important therefore to better understand the drivers for dual use and to understand whether dual use is a transitioning phase between cigarette smoking and complete smoking abstinence. Drivers of dual use may be a facet of the products themselves; that is, they do not currently generate nicotine delivery, sensorial acceptance, and subjective effects sufficient to facilitate complete transitioning, or a facet of their convenience and the ability to use them in places where smoking is prohibited such that when barriers to smoking are not present, cigarette smoking displaces HTP use. While clinical studies assessing nicotine pharmacokinetics and subjective effects suggest that these aspects of currently available HTPs should not be barriers to uptake in adult smokers [233-239], this is not being manifested at the population level. This could arise due to differences between clinical laboratory and real‑world use and potentially therefore a better understanding of nicotine delivery and subjective effects of HTPs in more naturalistic settings is warranted. In addition, the development of HTPs that better meet adult smokers’ needs may help increase uptake and transitioning and enhance the THR potential of HTPs.

A further area that warrants further examination is the perception of the risks associated with HTP use. In many studies, survey respondents expressed their perception that using HTPs was equally as harmful as, or more harmful than, cigarette smoking. Such a misperception does not align with the scientific literature, which is increasingly demonstrating significantly lower exposure to HPHCs and other toxicants, and therefore a potentially reduced risk profile, of HTPs relative to cigarettes [19,42-46]. While the reasons for these misperceptions are not abundantly clear, they appear to be becoming increasingly prevalent. What is perhaps clearer is that these misperceptions may be a barrier to the uptake of HTPs among adult smokers. Overall, this suggests a need, if the THR potential of HTPs is to be maximised, for better communication to adult smokers of the RR profile of HTPs and cigarettes to correct the misperceptions while increasing uptake among this population. Breaking down these barriers would necessitate educating not only smokers but also policymakers, regulators, and healthcare providers about the reduced harm potential of non-combustible nicotine delivery products and enable informed decision-making. The finding in this review that HTP use is more common among the younger adult population additionally suggests that proper risk communication could be targeted towards the remaining older adult population of smokers, among whom smoking rates are high and who are most at risk of smoking‑related disease [240].

One aspect of potential concern for any novel tobacco product is the possibility that it could act as a ‘gateway’ between non‑use of tobacco products and use initiation, and further towards initiation of cigarette smoking [48,92,126]. Several studies described in this review looked at the 'gateway' hypothesis, including the studies of Gottschlich et al. [125] and Bast et al. [108], which provided good evidence against the 'gateway' hypothesis of HTPs. However, these studies may have been limited by their cross‑sectional design, as longitudinal studies are better suited to explore any causal effects between the use of one tobacco product and the initiation of use of another. While no firm conclusions can therefore be made regarding whether HTPs do provide a 'gateway' into tobacco use and whether this mitigates the THR potential of HTPs, it is encouraging that the prevalence of ever use of HTPs among never smokers, and particularly among youth, was found to be extremely low in many of the studies reviewed. That said, it is important to constantly monitor whether HTPs are acting as a ‘gateway’ into tobacco/nicotine use, particularly as the use of HTPs becomes more widespread and as manufacturers market HTPs in an increasing number of countries.

It is worthwhile pointing out a limitation, both of this review and of many of the individual papers that we described. The majority of studies conducted on HTPs, particularly when assessing use prevalence and changes in behaviour, are both cross-sectional and observational in nature. From such studies, it is challenging to causally establish changes in both individual and population health as they are better suited to assessing associations. In order to address this limitation and to facilitate a better understanding of the potential health impacts of HTPs and their THR potential, longitudinal follow-up studies may be required to assess changes in smoking behaviour subsequent to HTP use initiation and to determine the causal existence of changes in both individual and population health.

## Conclusions

In summary, the positive individual‑level benefits of transitioning to using HTPs may also be seen at the population level. HTP use is almost exclusively found among those with a history of cigarette smoking, and there is good evidence of the ability of HTPs to provide a transitioning benefit among adult smokers and in the absence of any significant ‘gateway’ into tobacco/nicotine use, particularly among youth. Dual use of HTPs and cigarettes is frequently reported. When such use is concomitant with reductions in cigarette consumption, this may potentially contribute to THR by reducing overall toxicant exposure. Furthermore, a period of dual use may be part of the overall transitioning process. The nature of dual use (e.g., is it associated with reductions in cigarette consumption and/or future likelihood of complete transitioning or is it prolonging smoking among those intending to quit) warrants further investigation. In addition, correction of the widespread and increasing misperceptions of HTPs among adult smokers is recommended to promote HTP uptake among this population.
